# Impact of in vitro SARS-CoV-2 infection on breast cancer cells

**DOI:** 10.1038/s41598-024-63804-3

**Published:** 2024-06-07

**Authors:** Michele Sommariva, Maria Dolci, Tiziana Triulzi, Federico Ambrogi, Matteo Dugo, Loris De Cecco, Valentino Le Noci, Giancarla Bernardo, Martina Anselmi, Elena Montanari, Serenella M. Pupa, Lucia Signorini, Nicoletta Gagliano, Lucia Sfondrini, Serena Delbue, Elda Tagliabue

**Affiliations:** 1 Dipartimento di Scienze Biomediche per la Salute, Università degli Studi di Milano, Via Mangiagalli 31, 20133 Milan, Italy; 2 Microambiente e Biomarcatori dei Tumori Solidi, Dipartimento di Oncologia Sperimentale, Fondazione IRCCS Istituto Nazionale dei Tumori di Milano, Via Amadeo 42, 20133 Milan, Italy; 3 Dipartimento di Scienze Biomediche, Chirurgiche ed Odontoiatriche, Università degli Studi di Milano, Via Pascal 36, 20133 Milan, Italy; 4 Dipartimento di Scienze Cliniche e di Comunità, Università degli Studi di Milano, Via Celoria 22, 20133 Milan, Italy; 5 Department of Medical Oncology, IRCCS Ospedale San Raffaele, Via Olgettina 60, 20132 Milan, Italy; 6 Integrated Biology of Rare Tumors, Dipartimento di Oncologia Sperimentale, Fondazione IRCCS Istituto Nazionale dei Tumori di Milano, Via Amadeo 42, 20133 Milan, Italy

**Keywords:** SARS-CoV-2, Breast cancer, Estrogen receptor, Luminal A breast cancer, Cancer, Breast cancer, Tumour virus infections, Oncology, Cancer

## Abstract

The pandemic of coronavirus disease 19 (COVID-19), caused by severe respiratory syndrome coronavirus 2 (SARS-CoV-2), had severe repercussions for breast cancer patients. Increasing evidence indicates that SARS-CoV-2 infection may directly impact breast cancer biology, but the effects of SARS-CoV-2 on breast tumor cells are still unknown. Here, we analyzed the molecular events occurring in the MCF7, MDA-MB-231 and HCC1937 breast cancer cell lines, representative of the luminal A, basal B/claudin-low and basal A subtypes, respectively, upon SARS-CoV-2 infection. Viral replication was monitored over time, and gene expression profiling was conducted. We found that MCF7 cells were the most permissive to viral replication. Treatment of MCF7 cells with Tamoxifen reduced the SARS-CoV-2 replication rate, suggesting an involvement of the estrogen receptor in sustaining virus replication in malignant cells. Interestingly, a metagene signature based on genes upregulated by SARS-CoV-2 infection in all three cell lines distinguished a subgroup of premenopausal luminal A breast cancer patients with a poor prognosis. As SARS-CoV-2 still spreads among the population, it is essential to understand the impact of SARS-CoV-2 infection on breast cancer, particularly in premenopausal patients diagnosed with the luminal A subtype, and to assess the long-term impact of COVID-19 on breast cancer outcomes.

## Introduction

In the last 3 years, the coronavirus disease-19 (COVID-19) pandemic has emerged as the most relevant global health problem^1^. COVID-19 is caused by severe acute respiratory syndrome coronavirus 2 (SARS-CoV-2), which belongs to the *Coronaviridae* family of enveloped, single-stranded, positive-sense RNA viruses. Through the Angiotensin Converting Enzyme-2 (*ACE2*) receptor and Transmembrane serine protease 2 (*TMPRSS2*), SARS-CoV-2 can enter host cells and start its replication program. Although the primary target cells of this virus are the cells of the respiratory system, SARS-CoV-2 can infect, at least theoretically, all cells expressing the *ACE2* receptor^2,3^. Since this virus has spread worldwide, it had—and in some cases still has—profound negative repercussions on national health systems, especially in the management of patients with other pathologies, such as breast cancer^4,5^. Moreover, it has been proposed that breast cancer patients have a greater probability of severe outcomes after being infected with SARS-CoV-2, probably due to impairment of their immune function caused by both tumor cells themselves and anticancer therapies^6^. Montopoli et al. reported that women with hormone-driven breast and ovarian cancers are at increased risk of being infected with SARS-CoV-2 and developing more severe forms of COVID-19, as indicated by the greater rates of hospitalization and death in these patients than in noncancer patients^7^. Some lines of evidence suggest that SARS-CoV-2 infection may directly impact breast cancer progression and outcome. For instance, it has been reported that the SARS-CoV-2 M protein can exacerbate the malignant features of the MDA-MB-231 triple-negative breast cancer (TNBC) cell line, such as its migratory and metastatic potential^8^, while the SARS-CoV-2 spike (S) protein can increase the proliferation of estrogen receptor (ER)-positive MCF-7 cells^9^. However, a deep comprehension of the direct effects of SARS-CoV-2 on breast cancer cells has not been achieved yet.

Therefore, the aim of the present study was to comprehensively describe the molecular events occurring in breast cancer cell lines of three different molecular subtypes upon SARS-CoV-2 infection at gene and cellular levels. A better understanding of the intricate interconnections between breast cancer and COVID-19 represents an urgent clinical need in order to assess the long-term consequences of COVID-19 on breast cancer outcomes.

## Results

### Different permissiveness of breast cancer cell lines to SARS-CoV-2 infection

**Figure 1:**
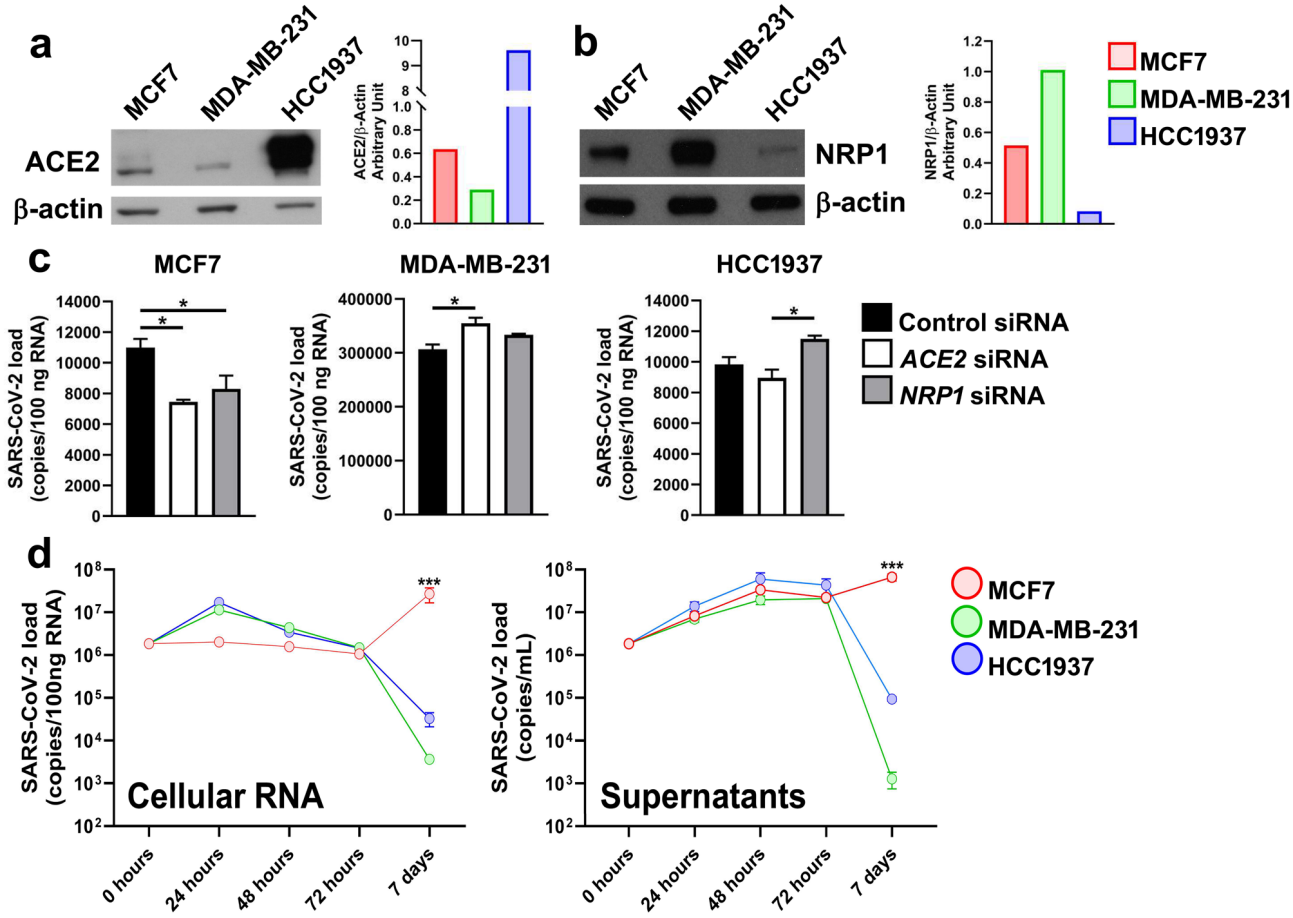
SARS-CoV-2 replication in breast cancer cell lines. Western blot and densitometric analysis of (**a**) ACE2 and (**b**) NRP1 protein expression in the MCF7, MDA-MB-231 and HCC1937 breast cancer cell lines. (**c**) *ACE2* and *NRP1* were silenced by siRNAs in the MCF7, MDA-MB-231 and HCC1937 breast cancer cell lines, which were subsequently infected with SARS-CoV-2 lineage B1. Viral RNA, expressed as copies/100 ng RNA, was quantified in the cytoplasm 6 h p.i. by real-time PCR with primers targeting the viral N1 gene. The data are presented as the means ± SEMs and are representative of one of three independent experiments with similar results. * *p* < 0.05 by one-way ANOVA followed by Tukey’s multiple comparison test with a single pooled variance. (**d**) MCF7, MDA-MB-231 and HCC1937 breast cancer cells were infected with SARS-CoV-2, and viral RNA was quantified at different time points in the cytoplasm and in the supernatants by real-time PCR. The viral load is expressed as copies/100 ng RNA or copies/mL for the cellular or supernatant RNA, respectively. The data are presented as the means ± SEMs and are representative of one of three independent experiments with similar results. *** *p* < 0.001 according to two-way ANOVA with multiple comparisons; refers to the comparison between MCF7 cells and MDA-MB-231 cells and the comparison between MCF7 cells and HCC1937 cells.

We first evaluated the expression level of *ACE2*, the main entry receptor for SARS-CoV-2^10^. By western blot analysis, we observed that HCC1937 cells expressed the *ACE2* protein at a higher level than did the other two cell lines (Fig. 1a), and these data were corroborated by immunofluorescence analysis (Supplementary Fig. S1). Neuropilin 1 (*NRP1*), another SARS-CoV-2 cellular binding factor^11^, was more abundant in MCF7 and MDA-MB-231 cells than in HCC1937 cells (Fig. 1b). According to densitometric analysis, in MCF7 cells, the expression of the two receptors was almost equal, while in the two other cellular models, the two receptors exhibited opposite expression patterns (Fig. 1a, b). To determine the importance of *ACE2* and *NRP1* in mediating SARS-CoV-2 entry into the three breast cancer cell lines, we silenced *ACE2* and *NRP1* using specific short interfering RNAs (siRNAs). The silencing efficiency was evaluated by western blotting (Supplementary Fig. S2). Control, *ACE2*-silenced and *NRP1*-silenced cells were then infected with SARS-CoV-2, lineage B.1, and the viral load was assessed by real-time PCR 6 h post-infection (p.i.). Silencing of either *ACE2* or *NRP1* reduced viral replication in MCF7 cells but not in MDA-MB-231 and HCC1937 cells (Fig. 1c).

Subsequently, we monitored SARS-CoV-2 replication in the breast cancer cell cytoplasm at different time points. The RNA copy number in the MDA-MB-231 and HCC1937 cell lines peaked 24 h p.i., after which it gradually decreased over time. In MCF7 cells, viral RNA, whose level remained stable during the first 72 h after SARS-CoV-2 infection, greatly increased between the third and seventh days post infection (Fig. 1d). Quantification of viral RNA in the cell supernatants revealed a progressive increase in the viral RNA copy number in all three cell lines during the first 3 days after infection. At day 7, the amount of viral RNA had abruptly decreased in MDA-MB-231 and HCC1937 cells, while in MCF7 cells, it had increased further, mirroring the pattern observed within the cytoplasm (Fig. 1d).

### Effect of SARS-CoV-2 infection on breast cancer cell viability and motility

**Figure 2:**
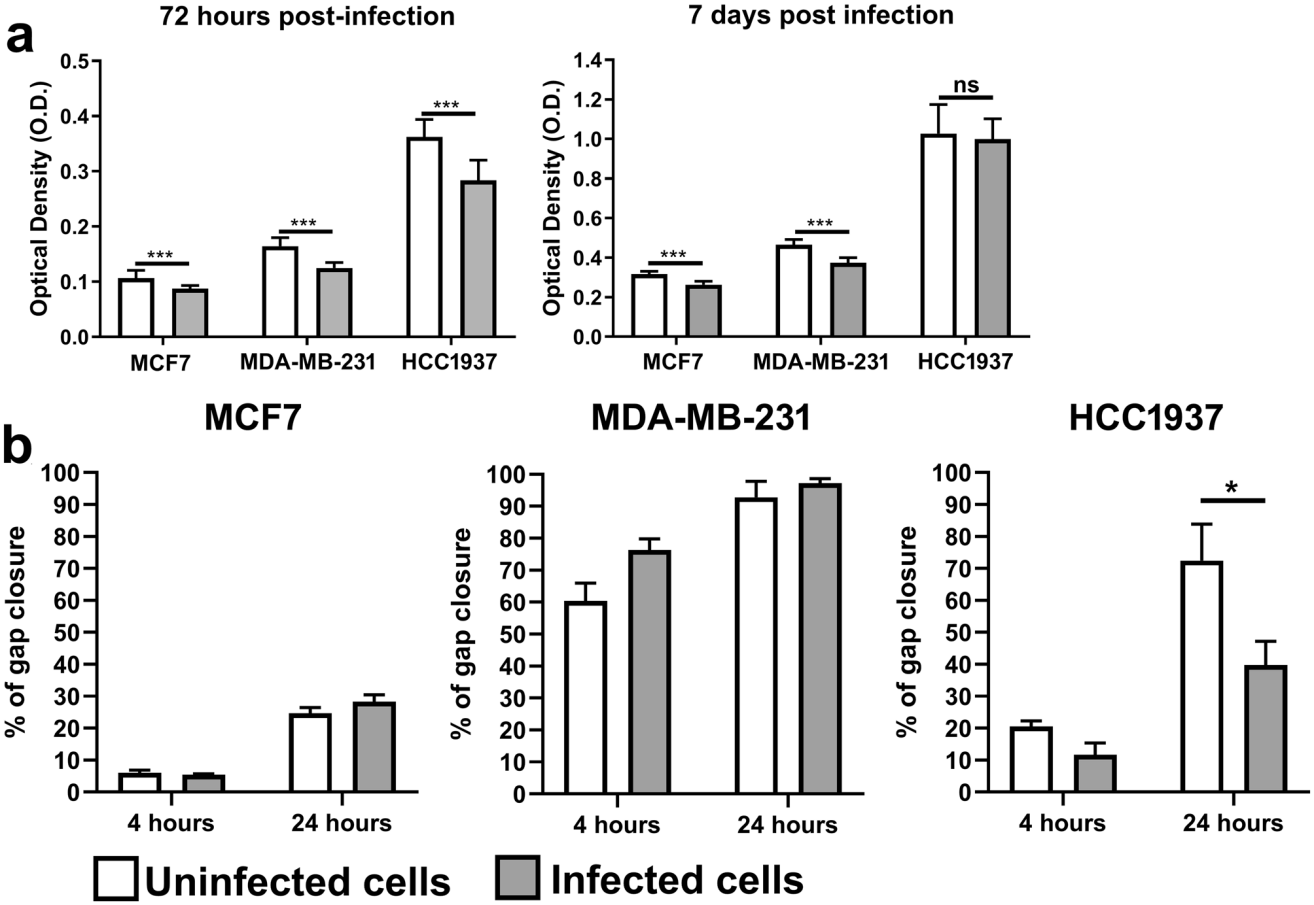
Effect of SARS-CoV-2 infection on breast cancer cell proliferation and motility. (**a**) MTT proliferation assay of MCF7, MDA-MB-231 and HCC1937 breast cancer cells after 72 h or 7 days of SARS-CoV-2 infection. Uninfected cells served as controls. The data are presented as the means ± SEMs and are representative of one of three independent experiments with similar results. ***p < 0.001 by unpaired two-tailed t test. (**b**) Confluent monolayers of MCF7, MDA-MB-231 and HCC1937 cells were infected with SARS-CoV-2. After 2 h of infection, the viral inoculum was removed, and gap wounds were generated by mechanical scratching. Cell migration into the wounded area was observed at 0, 4 and 24 h p.i. Histograms showing the extent of wound closure at the indicated time points are presented, with data shown as the percentages of wound closure at a specific time point compared to 0 h. The data are presented as the means ± SEMs and are representative of one of three independent experiments with similar results. *p < 0.05 by one-way ANOVA followed by Tukey’s multiple comparison test with a single pooled variance.

We next evaluated the cytotoxic effect of SARS-CoV-2 on breast cancer cells by an MTT assay. A reduction of approximately 20% in cell proliferation was observed in all the cell lines 72 h after SARS-CoV-2 infection (-17.84%, -24.12%, and -21.73% in the infected MCF7, MDA-MB-231 and HCC1937 cell lines, respectively, compared to their uninfected counterparts). Seven days post infection, a similar trend was maintained only for infected MCF7 and MDA-MB-231 cells (-16.78% and -19.48%, respectively, compared to the corresponding controls) (Fig. 2a). A negligible effect on cancer cell viability was also confirmed by colony formation assays carried out 14 days after SARS-CoV-2 infection (Supplementary Fig. S3). Furthermore, SARS-CoV-2 did not appear to interfere with the motility of cancer cells except for infected HCC1937 cells, which showed a reduced migratory capacity compared to that of the uninfected control cells at 24 h p.i. (Figs. 2b, S3).

### Effect of SARS-CoV-2 infection on the breast cancer cell gene expression profile

**Figure 3:**
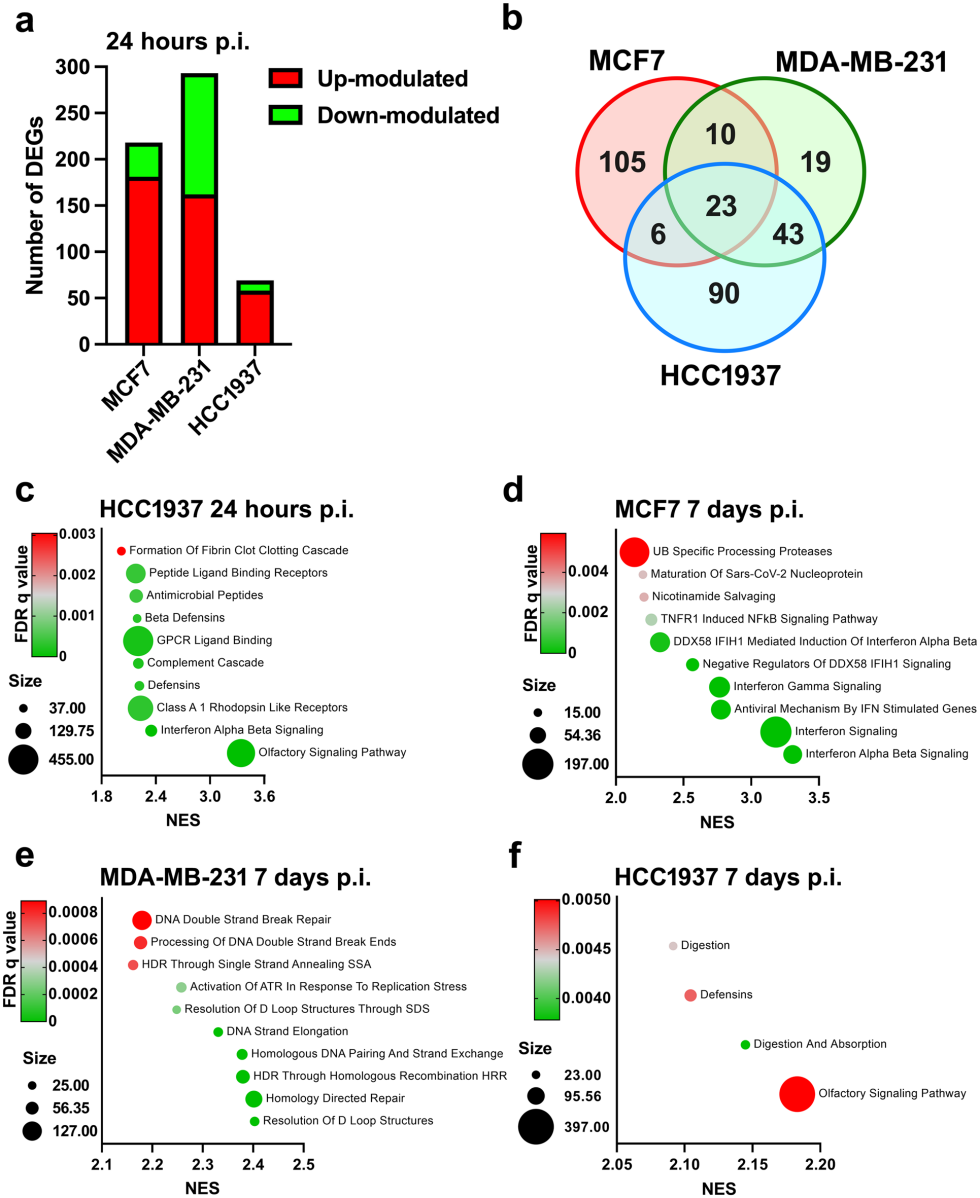
Effect of SARS-CoV-2 infection on breast cancer cell gene expression profiles. MCF7, MDA-MB-231 and HCC1937 breast cancer cells were infected with SARS-CoV-2 lineage B1, and gene expression profile analysis was performed at 24 h and 7 days p.i. (**a**) Number of DEGs (FDR < 0.05) identified by the comparison of infected and control cells of each cell line 24 h p.i. (**b**) Overlapping upregulated DEGs (FDR < 0.05) identified by the comparison of infected and control cells of each cellular model 24 h p.i. (**c-e**) Bubble plots of the top 10 Reactome pathways with an FDR q-value < 0.05 found to be enriched in infected cells versus uninfected cells as determined by preranked GSEA. (**f**) Bubble plot of the only 4 Reactome pathways with an FDR q-value < 0.05 found to be enriched in infected cells versus uninfected HCC1937 cells at 7 days p.i., as determined by preranked GSEA. The X-axis shows the normalized enrichment score (NES). The bubble area is proportional to the size of the gene set, and the bubble color represents the FDR q value.

The influence of viral infection on the breast cancer cell gene expression profile was investigated by microarray analysis carried out 24 h and 7 days p.i. At the earliest time point, SARS-CoV-2 infection had the strongest effect on the MDA-MB-231 cell line (293 Differentially Expressed Genes, DEGs; 162 upregulated and 131 downregulated), followed by the MCF7 cell line (218 DEGs; 181 upregulated and 37 downregulated). In HCC1937 cells, the influence of infection was weaker, resulting in only 69 DEGs (58 upregulated and 11 downregulated) (Fig. 3a and Supplementary Table S1). We determined the intersection of the upregulated genes among the three breast cancer cell lines and identified 23 overlapping DEGs, with no overlapping downregulated DEGs (Fig. 3b). Interestingly, 12 of the 23 (52.17%) overlapping upregulated DEGs belonged to the olfactory receptor family (Supplementary Table S2). Correlation analysis between the expression levels of these 23 genes and the viral load revealed a statistically significant correlation only for the ADAMTS19 gene; however, the correlation was positive in MCF7 cells but negative in the other two cell lines, due to the inter-cell line variability (Supplementary Fig. S4). At 7 days p.i., SARS-CoV-2 infection no longer influenced the transcriptome profile of the MDA-MB-231 and HCC1937 cells (1 and 0 DEGs, respectively), while a marginal effect was still observed in the MCF7 cell line (30 DEGs; 28 upregulated and 2 downregulated genes) (Supplementary Table S3). Metascape analysis of the 28 upregulated DEGs indicated that these genes were mostly involved in viral recognition and the antiviral immune response and, notably, in the SARS-CoV-2 signaling pathway (Supplementary Fig. S5).

Functional analysis via Gene set enrichment analysis (GSEA) revealed that the olfactory signaling pathway was the only significantly enriched biological pathway in infected MCF7 and MDA-MB-231 cells 24 h p.i. In contrast, almost all the pathways enriched in HCC1937 cells exposed to the SARS-CoV-2 were involved in innate pathogen recognition and immune activation (Fig. 3c). At 7 days p.i., the findings were completely opposite. According to the virus titration data, in infected MCF7 cells, several pathways associated with coronavirus infection were active. We also detected gene sets clearly involved in viral recognition and immune activation and response (Fig. 3d). Interestingly, when we expanded the list of gene sets to all those with an False Discovery Rate (FDR) q value < 0.25, the value indicated by the GSEA developers as the default cutoff, we found 3 ER-related gene pathways (Estrogen-dependent gene expression; ESR-mediated signaling; Estrogen-dependent nuclear events downstream of ESR membrane signaling) (data not shown). In the MDA-MB-231 cell line, SARS-CoV-2 seemed to drive the activation of DNA repair processes and cell cycle progression (Fig. 3e). Finally, in HCC1937 cells, only four gene sets, mostly related to digestion and absorption, met the statistical threshold, and only one immune gene set remained (Fig. 3f). The complete list of statistically significant pathways is provided in Supplementary Table S4.

### Impact of the SARS-CoV-2 metagene on breast cancer patient survival

**Figure 4:**
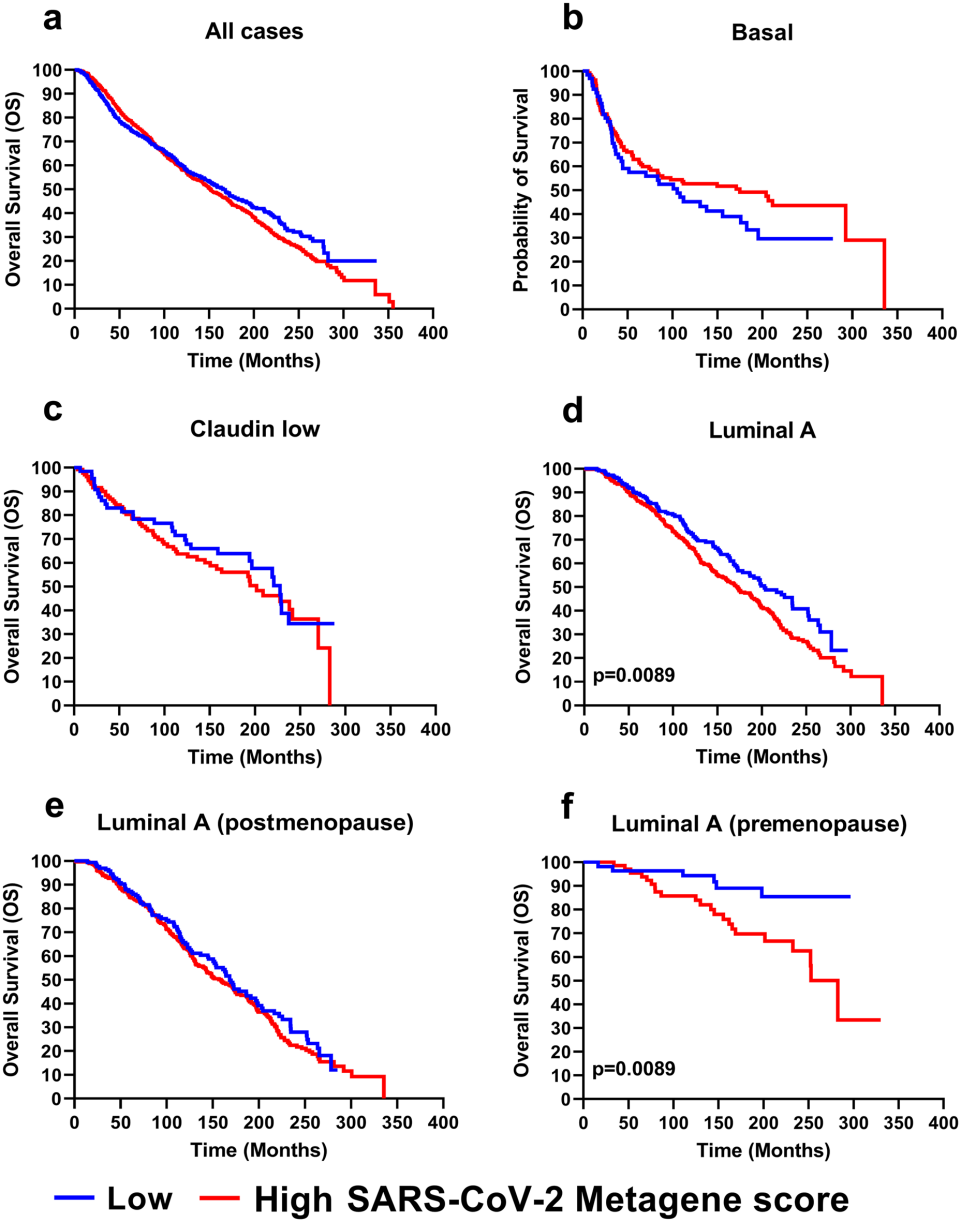
Impact of the SARS-CoV-2 Metagene on breast cancer patient survival. Breast cancer patients represented in the METABRIC dataset were stratified as having a high (red line) or a low (blue line) SARS-CoV-2 Metagene score, with the lower tertile used as the cutoff. Kaplan–Meier curves of overall survival (OS) for all patients (**a**), the basal subgroup (**b**), the claudin-low subgroup (**c**), the luminal A subgroup (**d**) and the postmenopausal (**e**) and premenopausal (**f**) luminal A breast cancer subgroups in the METABRIC dataset. Differences between groups were estimated by the log-rank test.

To evaluate whether genes modulated by SARS-CoV-2 infection may have an impact on breast cancer patient survival, the abovementioned 23 genes were utilized to generate a “SARS-CoV-2 Metagene”, which was subsequently applied for analysis of the METABRIC dataset. The patients were stratified as having a “high” or “low” SARS-CoV-2 Metagene score, with 30.67% (n = 584) and 69.33% (n = 1320) patients, respectively, in each group. Kaplan–Meier analysis of the patients for whom survival information was available did not reveal any statistically significant difference in terms of overall survival (OS) between the two groups (Fig. 4a). Similarly, intrinsic molecular subgroup analysis revealed no differences in the survival of basal B/claudin-low or basal A patient survival (Fig. 4b, c). However, luminal A breast cancer patients, whose clinical characteristics are detailed in Supplementary Table S5, with a high SARS-CoV-2 Metagene score were characterized by significantly poorer OS than those with a low metagene score (Fig. 4d). Multivariate analysis incorporating clinical predictors with a *p* value < 0.1 in univariate analysis, highlighted the lack of a significant association between the SARS-CoV-2 Metagene and OS (Table 1). However, a multivariable model considering an interaction term between menopausal status and the SARS-CoV-2 Metagene showed a statistically significant interaction (*p* = 0.028) (Table 2). Although in postmenopausal patients, there was no evidence of an effect of SARS-CoV-2 Metagene (HR = 1.09; 95% CI: 0.85–1.39), in premenopausal patients, the effect was statistically significant (HR = 3.12; 95% CI: 1.26–7.76) (Fig. 4e, f).

**Table 1:** Univariate and multivariate analyses of overall survival in luminal A breast cancer patients in the METABRIC dataset.

Variable	Univariate analysis			Multivariate analysis		
	HR	CI 95%	*p* value	HR	CI 95%	*p* value
SARS-CoV-2 Metagene (High vs. Low)	1.354	1.078–1.701	0.009	1.187	0.935–1.507	0.159
Lymph Node Status (Positive vs. Negative)	1.544	1.255–1.900	< 0.001	1.233	0.942–1.614	0.127
Tumor Grade (G2 + G3 vs. G1)	1.324	0.980–1.788	0.067	1.174	0.866–1.592	0.302
Tumor Size (T2 + T3 vs. T1)	1.702	1.382–2.097	< 0.001	1.584	1.270–1.977	< 0.001
Hormone Therapy (Therapy vs. No therapy)	1.623	1.294–2.035	< 0.001	1.057	0.785–1.424	0.715
Inferred menopause (Post vs. Pre)	3.739	2.541–5.503	< 0.001	3.419	2.294–5.095	< 0.001

Univariate and multivariate analyses of overall survival in luminal A breast cancer patients (n = 679) represented in the METABRIC dataset. Multivariate Cox proportional hazards regression analysis was performed with clinical predictors with a *p* value < 0.1 in univariate analysis. HR: Hazard ratio estimated by the Cox proportional hazards regression model; CI: Confidence interval of the estimated HR; G: Tumor grade; T: Tumor size. The *P* values were calculated by Cox proportional hazards regression analysis.

**Table 2:** Multivariate analysis of luminal A breast cancer patients in the METABRIC dataset.

Variable	Multivariate analysis		
	HR	CI 95%	*p* value
SARS-CoV-2 Metagene (High vs. Low)	3.12	1.26–7.76	0.01
Lymph Node Status (Positive vs. Negative)	1.23	0.94–1.62	0.12
Tumor Grade (G2 + G3 vs. G1)	1.16	0.85–1.57	0.35
Tumor Size (T2 + T3 vs. T1)	1.59	1.27–1.98	< 0.001
Hormone Therapy (Therapy vs. No therapy)	1.06	0.78–1.42	0.71
Inferred menopause status (Post vs. Pre)	20.19	3.64–112	< 0.001
Inferred menopause status by SARS-CoV-2 Metagene	0.35	0.14–0.89	0.028

Multivariate analysis of luminal A breast cancer patients (n = 679) represented in the METABRIC dataset revealed an interaction between the inferred menopause status and the SARS-CoV-2 Metagene. HR: hazard ratio estimated by the Cox proportional hazards regression model; CI: confidence interval of the estimated HR; G: Tumor grade; T: Tumor size. The *P* values were calculated by Cox proportional hazards regression analysis.

### Role of the estrogen receptor in SARS-CoV-2 infection

**Figure 5:**
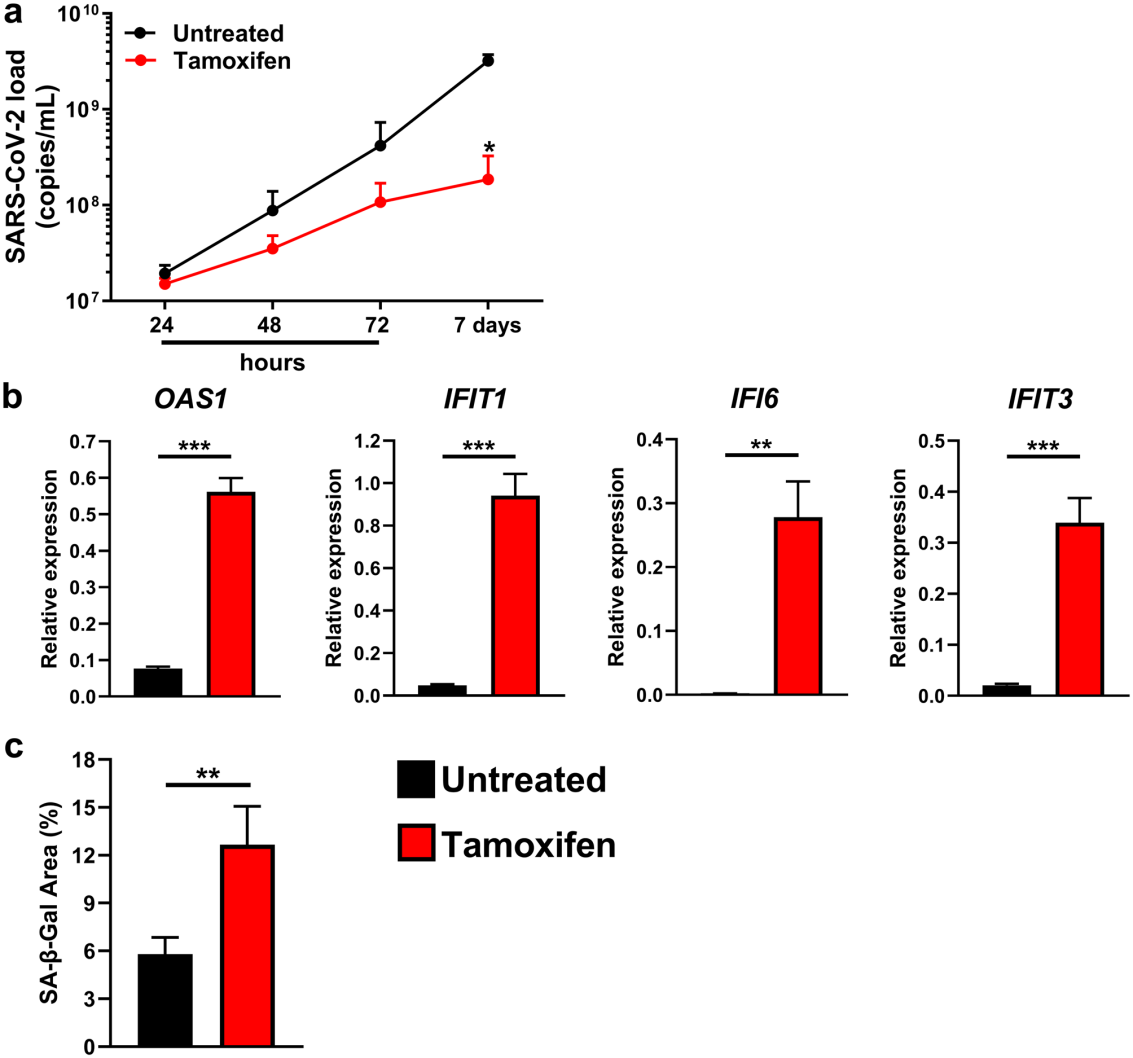
Effect of Tamoxifen treatment on SARS-CoV-2 replication in MCF7 cells. SARS-CoV-2-infected MCF7 cells were exposed to 10 µM Tamoxifen 24 h before infection, at the time of infection, immediately post infection and throughout the experiment. (**a**) The viral load in the supernatants was evaluated at 24, 48, and 72 h and at 7 days p.i. by real-time PCR. The data, expressed as copies/mL, are presented as the means ± SEMs and are representative of one of three independent experiments with similar results. *p < 0.05 by two-way analysis of variance with multiple comparisons. (**b**) Real-time PCR analysis of *OAS1*, *IFIT1*, *IFI6*, and *IFIT3* gene expression was performed on mRNA extracted from the samples collected at 7 days p.i. described in (**a**). The results are presented as 2^−ΔCt^ values. ** *p* < 0.01, *** *p* < 0.001 by two-tailed unpaired Student’s t test. (**c**) MCF7 cells were infected and exposed to 10 µM Tamoxifen throughout the experiment. Seven days p.i., the cells were stained for determination of senescence-associated β-galactosidase (SA-β-Gal) activity. The histograms show the percentage of the SA-β-Gal-positive area evaluated in four randomly selected fields for each sample acquired with constant parameters. The data are presented as the means ± SEMs. **p < 0.01 by the Mann–Whitney U test.

The bioinformatic and survival analyses highlighted the possible involvement of ER in SARS-CoV-2 infection. Therefore, we investigated whether pharmacological inhibition of ER can counteract SARS-CoV-2 replication in ER-positive MCF7 cells^12^, which are characterized by the highest permissiveness to the virus (Fig. 1d). The selected dose of Tamoxifen (10 μM) effectively dampened ER activity, as demonstrated by the reductions in the BCL2 Apoptosis Regulator (BCL-2) and Progesterone Receptor (PGR) mRNA levels^13,14^ and inhibited MCF7 cell proliferation in a time-dependent manner (Supplementary Fig. S6). Subsequently, SARS-CoV-2-infected MCF7 cells were exposed to Tamoxifen 24 h before SARS-CoV-2 infection, at the time of infection or immediately after infection. Quantification of the viral load did not reveal any significant difference between Tamoxifen-treated and untreated cells (Supplementary Fig. S6). However, the presence of Tamoxifen throughout the experiment had a strong inhibitory effect on SARS-CoV-2 replication, with a statistically significant (*p* = 0.025) difference at 7 days p.i. (Fig. 5a). This finding was also confirmed by a plaque reduction assay carried out on VERO E6 cells, which showed a plaque reduction of 72.6% (PFU: plaque-forming unit. 5.48 × 10^3^ PFU/mL and 1.50 × 10^3^ PFU/mL for untreated and Tamoxifen-treated infected MCF7 cells, respectively). Interestingly, we also found that the mRNA levels of 2′-5′-Oligoadenylate Synthetase 1 (*OAS1*), Interferon Induced Protein with Tetratricopeptide Repeats 1 (*IFIT1*), Interferon Alpha Inducible Protein 6 (*IFI6*), and Interferon Induced Protein with Tetratricopeptide Repeats 3 (*IFIT3*), the top 4 upregulated genes in infected MCF7 cells 7 days p.i. (Supplementary Table S3), were further increased in Tamoxifen-treated cells (Fig. 5b). SARS-CoV-2 and Tamoxifen are reported to promote cell senescence^15,16^. Since senescence can be utilized by cells to restrict viral replication^17,18^ and GSEA of our gene expression profile data showed that at 7 days p.i., there was statistically significant enrichment of a gene set related to senescence (Oncogene-induced senescence) in infected MCF7 cells compared to the controls (Supplementary Table S4), we investigated whether the reduction in viral load observed in Tamoxifen-treated and infected MCF7 cells could be ascribed to induction of the senescence program. We performed senescence-associated-β-galactosidase (SA-β-Gal) staining, a method widely utilized to assess cellular senescence^19^. MCF7 cells were infected with SARS-CoV-2 and treated with or without Tamoxifen, as described above. In infected cells at 7 days p.i., the intensity of SA-β-Gal staining increased in response to Tamoxifen exposure (Figs. 5c and S6).

## Discussion

In the present work, we evaluated the biological effect of SARS-CoV-2 infection on MCF7 cells (*ER*^+^/*PGR*^+^), a model for the luminal A subtype, MDA-MB-231 and HCC1937 cell lines (*ER*^-^/*PGR*^-^), collectively referred to as triple-negative breast cancer (TNBC) and belonging to the basal B/claudin low and basal A subtypes, respectively^12^. Although these three cell lines do not cover the entire complexity of the molecular breast cancer subtype landscape^20^, we decided to focus our efforts only on the most frequent (Luminal A) and the most aggressive (TNBC) breast cancer phenotypes^21,22^.

Considering the different expression patterns in our cellular models, we first investigated the involvement of *ACE2* and *NRP1* in SARS-CoV-2 entry by silencing experiments^10,11^. In MCF7 cells, silencing of either *ACE2* or *NRP1* reduced the viral load, suggesting that these two receptors play important roles in mediating SARS-CoV-2 entry in this cell line. However, viral infection did not seem to be particularly affected by reduced levels of the *ACE2* and *NRP1* proteins in the MDA-MB-231 and HCC1937 cell lines. These dissimilar behaviors may be explained by the presence of alternative receptors that can facilitate SARS-CoV-2 entry into cells as well as its propagation through a cell-to-cell transmission mechanism^23,24^.

We then monitored the kinetics of SARS-CoV-2 replication in breast cancer cell lines and observed that MCF7 cells showed the highest permissiveness to viral replication. Accordingly, pathway analysis revealed enrichment of SARS associated coronaviruses-related gene sets in infected MCF7 cells (i.e., maturation of SARS-CoV-2 nucleoprotein, SARS-CoV-1 infection, translation of SARS-CoV-1 structural proteins, SARS-CoV-2 activates/modulates innate and adaptive immune responses, and SARS-CoV-2 infection), supporting the observation of active replication of the virus even 7 days after the initial infection. Moreover, we detected enrichment of ER-related pathways in infected MCF7 cells at 7 days p.i., when viral replication peaked, possibly suggesting that ER signaling is more active in infected cells.

It has been reported that the SARS-CoV-2 spike (S) protein, by binding to ER and modulating its biological activity, can increase MCF7 cell proliferation and that this effect is abrogated by treatment with Raloxifene, a selective estrogen receptor modulator (SERM)^9,25^. However, our data showed a slight decrease in the proliferation of infected MCF7 cells compared to their uninfected counterparts. This discrepancy may be explained by the fact that the reported increase in cell growth was obtained by exposing tumor cells to purified SARS-CoV-2 S protein and not to a complete replicating virus, which, unlike isolated proteins, can induce cellular stress, proliferation arrest and senescence^16,26,27^. Accordingly, one of the gene-sets significantly enriched in infected MCF7 cells at 7 days p.i. was related to senescence (oncogene-induced senescence), possibly suggesting that this phenomenon can occur in the MCF7 cell line at a late stage of infection. In addition, we also detected enrichment of several pathways associated with coronavirus infection and gene sets clearly involved in viral recognition and immune activation in infected MCF7 cells at 7 days p.i. It may be possible that, throughout the first week of infection, MCF7 cells are not able to properly recognize the virus or mount an efficacious antiviral response. When the viral load reaches a considerable concentration, cells begin to activate several defense mechanisms against the virus, including senescence^17,18^. Interestingly, the four genes most strongly upregulated in infected MCF7 cells compared to uninfected cells, namely, *OAS1*, *IFIT1*, *IFI6*, and *IFIT3*, belong to the Interferon-stimulated gene (ISG) family and are involved in viral replication restriction^28–30^.

The physical and functional crosstalk between the SARS-CoV-2 S protein and ER^9^ may represent a strategy utilized by the virus to sustain its replication and spread. This idea is supported by our data showing that inhibition of ER activity by Tamoxifen significantly reduced the viral load in MCF7 cells, which is in line with clinical observations. Indeed, it was reported that antiestrogen therapy reduced the incidence of COVID-19 in breast and ovarian cancer patients^7^ and contributed to SARS-CoV-2 infection resistance in breast cancer patients^31^. However, it should be highlighted that the precise mechanism by which ER-blockers function in restraining viral replication has not been fully elucidated. In addition to their ER-inhibitory activity, these drugs may exert anti-SARS-CoV-2 activity through ER-independent mechanisms, such as by blocking viral entry or inhibiting viral RNA synthesis^31^. It is unlikely that this drug can interfere with viral entry in MCF7 cells, since the experimental group treated with Tamoxifen at the time of SARS-CoV-2 infection exhibited viral replication kinetics overlapping those of the control group. Therefore, the observation that Tamoxifen-treated infected cells showed a reduced viral load may suggest the hypothesis that, at least in our cellular model, Tamoxifen may act in three different ways. As already mentioned, the first way involves inhibition of the biological activity of the estrogen receptor, which is presumably exploited by SARS-CoV-2 for its replication. The second proposed way is based on our findings showing that the intensity of β-Gal staining, a senescence marker^19^, was increased by Tamoxifen exposure. Although Tamoxifen is reported to be a senescence inducer ^15^, the observed increase cannot be attributable only to its pharmacological effect. Indeed, preliminary experiments carried out in the MCF7 cell line showed that tamoxifen treatment only slightly increased the proportion of senescent cells compared to that in the untreated control group (data not shown). Therefore, this ER-blocker may trigger cellular senescence, which, in turn, can function as an antiviral defense mechanism^17,18^. Although there is no consensus on this matter, the third possible way might rely on the intrinsic ability of Tamoxifen to directly induce the expression of genes with antiviral activity, such as ISGs^32,33^. We found that the expression levels of the top 4 genes upregulated by SARS-CoV-2 infection in MCF7 cells (*OAS1*, *IFIT1*, *IFI6*, and *IFIT3*) were further increased upon Tamoxifen exposure. Since these genes are involved in viral replication restriction^28–30^, we can speculate that Tamoxifen may boost the activity of signaling pathways already activated in the cell by the virus, eventually resulting in a more robust cellular immune response.

The SARS-CoV-2 titration data also revealed that the HCC1937 and MDA-MB-231 cell lines did not provide an optimal cellular environment for viral replication since the viral load in these cells decreased over time. Indeed, at 24 h p.i., HCC1937 cells were characterized by enrichment of immune-related pathways, possibly suggesting that these cells are trying to counteract the viral infection. However, in the MDA-MB-231 cell line, the virus did not appear to influence any specific pathways at 24 h p.i., but at the latest tested time point, there was enrichment of gene sets associated with DNA repair mechanisms and cell cycle progression, probably in response to DNA damage caused by SARS-CoV-2 infection, which may eventually lead to senescence and the elicitation of antiviral cellular immunity, as suggested^16^. Collectively, our observations indicate that the three cell lines showed different responses to SARS-CoV-2 infection, and we believe that the intrinsic characteristics of these cell lines may be the reason underlying their dissimilar permissivity to viral replication. First, the lack of ER expression in MDA-MB-231 and HCC1937 cells may corroborate the hypothesis that this protein is involved in supporting the SARS-CoV-2 life cycle. Second, as demonstrated by PROGENy analysis^34^, the MDA-MB-231 and HCC1937 cell lines are characterized by strong upregulation of the JAK/STAT pathway compared to that in the MCF7 cell line (z scores of -1.732, 1.536, and 2.637 for the MCF7, MDA-MB-231 and HCC1937 cell lines, respectively; data retrieved from https://www.proteinatlas.org/humanproteome/cell+line/Breast+cancer). Since the JAK/STAT pathway is relevant for regulating the response to viral infection^35^, the enrichment of this pathway in MDA-MB-231 and HCC1937 cells may explain why viral replication is efficiently restricted in these cell lines. Third, based on our microarray data, compared with MCF7 cells, MDA-MB-231 and HCC1937 cells are characterized by increased levels of Toll-like receptor 3 (*TLR3*), the RNA sensor RIG-I (*RIG-I*) and interferon induced with helicase C domain 1 (*MDA-5*), which are innate immune receptors involved in SARS-CoV-2 recognition^36^ (Supplementary Fig. S7). This distinctiveness may endow MDA-MB-231 and HCC1937 cells with increased sensitivity to viruses, with a consequent increased possibility of activation of defense mechanisms, which eventually limits viral replication.

Despite the high intercellular heterogeneity, our gene expression profile analysis revealed 23 overlapping upregulated genes among the three cell lines at 24 h p.i., suggesting that the virus can exert, at least in part, similar effects on these breast cancer cells.

Interestingly, several genes belonging to such gene list were associated with estrogen receptor signaling. For example, olfactory receptors (ORs), which regulate the proliferation, invasion, and metastasis of breast tumor cells^37^, thus affecting breast cancer patient survival^38^, may “dialog” with the estrogen receptor. Indeed, estradiol has been demonstrated to have a strong impact on the responsiveness of the olfactory epithelium to odorants^39^, and estrogen signaling appears to be important during the embryonic development of the olfactory bulb^40^. Some natural and synthetic odorant molecules are also reported to activate both ORs and the estrogen receptor^41^. Furthermore, one of the products of alternative splicing of MECOM, another of the 23 commonly up-regulated genes, and the ER pathways are characterized by several points of convergence^42^. Although connections between the other genes of the metagene signature and estrogen receptor signaling have not yet been identified, these genes may actively participate in shaping cancer cell behavior. For example, cadherin 18 (CDH18), which is highly expressed in the metastatic breast cancer cell line BrM2, may be involved in mammary tumor metastatic spread^43^, while the role of serine/threonine kinase 31 (STK31) in breast cancer development has been evaluated in animals and humans^44^.

The notion of a possible interconnection between the 23 up-regulated genes and the estrogen receptor may also be supported by our survival analysis. Indeed, the SARS-CoV-2 Metagene signature, generated by utilizing the above-mentioned 23 genes, was able to discriminate breast cancer patients with different OS only in the premenopausal luminal A subgroup, suggesting that the coordinated biological functions of these genes may impact breast cancer biology and clinical outcomes only when estrogen receptor signaling is active. In line, the metagene signature when applied to patients with TNBC (basal and claudin low), a subgroup associated to younger age and premenopausal status but negative for estrogen receptor expression^45,46^, did not show any prognostic value.

Previously published studies have reported the potential impact of SARS-CoV-2 infection in modulating breast cancer cell behavior^8,9^ and, in the present study, we contributed to expand the knowledge in this poorly understood field. We are aware that our in vitro experimental models, limited to only few breast cancer molecular subtypes, oversimplify the complex situation occurring in vivo during SARS-CoV-2 infection and do not take into consideration the effects of the virus on the tumor microenvironment and the role played by the immune system. However, our data can have important clinical relevance. Since we found that the estrogen receptor breast cancer cell line MCF7 was the most permissive to virus replication, it would be possible to imagine a scenario in which estrogen receptor positive breast tumors may act as a sort of SARS-CoV-2 reservoir that can continuously supplies new viral particles thus exacerbating COVID-19 aggressiveness and the consequences associated to this disease.

Although the vaccination dramatically reduced SARS-CoV-2 transmission^47^, this virus still spreads among the population, especially in vulnerable individuals such as cancer patients, and its effects on the health of breast cancer patients will only be assessable in the years ahead. Showing that the genes modulated by SARS-CoV-2 infection characterize a subgroup of luminal A breast cancer patients who experience worse outcomes, we want to emphasize the need to monitor the long-term impact of COVID-19 on breast cancer outcomes, particularly in premenopausal women.

Overall, this preclinical study provides interesting insights about the potential implications of SARS-CoV-2 infections on breast cancer cells but further studies are required to achieve a more definitive understanding of these effects.

## Methods

### Cell lines

MCF7 (HTB-22), MDA-MB-231 (CRM-HTB-26), and HCC1937 (CRL-2336) human breast cancer cells and VERO E6 normal monkey kidney cells (CRL-1586) were purchased from ATCC (American Type Culture Collection, Manassas, VA, USA). The breast cancer cell lines were maintained in RPMI 1640 medium supplemented with GlutaMAX™ (Gibco, Thermo Fisher Scientific, Waltham, MA, USA) and 10% fetal bovine serum (FBS; Gibco, Thermo Fisher Scientific, Waltham, MA, USA) at 37 °C in a 5% CO_2_ atmosphere. VERO E6 cells were maintained in DMEM supplemented with GlutaMAX™ (Gibco, Thermo Fisher Scientific, Waltham, MA, USA), 10% fetal bovine serum (FBS; Gibco, Thermo Fisher Scientific, Waltham, MA, USA) and 1% penicillin/streptomycin (Thermo Fisher Scientific, Waltham, MA, USA) at 37 °C in a 5% CO_2_ atmosphere. Within the last 3 years, the human cell lines had been authenticated using short tandem repeat profiling. All experiments were performed with mycoplasma-free cells, as determined with a MycoAlertTM PLUS Mycoplasma Detection Kit (Lonza, Basel, Switzerland).

### SARS-CoV-2 infection of breast cancer cell lines

MCF7, MDA-MB-231 and HCC1937 cells were infected with SARS-CoV-2, lineage B.1 (SARS-CoV-2/human/ITA/Milan-UNIMI-1/2020, GenBank MT748758.1), which was previously isolated in our laboratory ^48^. Cell infection with this virus was performed by viral adsorption in complete medium for 2 h using a multiplicity of infection (MOI) of 0.1 (8.78 × 10^8^ copies/mL) at 37 °C in a 5% CO_2_ atmosphere. All studies involving SARS-CoV-2 were performed in a Biosafety Level 3 laboratory.

### Western blot analysis

MCF7, MDA-MB-231 and HCC1937 cells were seeded at a density of 10^6^ cells/well in a 6-well culture plate (Thermo Fisher Scientific, Waltham, MA, USA) in complete RPMI 1640 medium and cultured at 37 °C in a humidified atmosphere containing 5% CO_2_. The following day, the cells were directly lysed with TNTG buffer (50 mM Tris–HCl (pH 7.5), 150 mM NaCl, 100 mM NaF, 10 mM sodium pyrophosphate, 10% (vol/vol) glycerol, 1% Triton X–100) supplemented with cOmplete Mini Protease Inhibitor Cocktail (Merck KGaA, Darmstadt, Germany) and 2 mM activated orthovanadate (Merck KGaA, Darmstadt, Germany). Western blot analysis was then carried out as described previously^49^. The recombinant rabbit monoclonal anti-ACE2 antibody (clone SN0754, dilution 1:1000, Thermo Fisher Scientific, Waltham, MA, USA), rabbit monoclonal anti-Neuropilin-1 (NRP1) antibody (clone D62C6, dilution 1:1000, Cell Signaling Technology Inc., Danvers, MA, USA) and horseradish peroxidase-conjugated mouse monoclonal anti-β-Actin antibody (clone AC-15, dilution 1:30,000, Merck KGaA, Darmstadt, Germany) served as primary antibodies. Semi-quantitative analysis of the Western blot data was performed by Quantity One 1-D Analysis Software (Bio-Rad Laboratories Inc., Hercules, CA, USA).

### ACE2 and NRP1 silencing

MCF7, MDA-MB-231 and HCC1937 cells were seeded at a density of 7 × 10^5^ cells/well in a 6-well culture plate (Thermo Fisher Scientific, Waltham, MA, USA) in complete RPMI 1640 medium and cultured at 37 °C in a humidified atmosphere containing 5% CO_2_. ACE2 and NRP1 were silenced using 50 nM *ACE2* or *NRP1* SMARTpool ON-TARGETplus siRNA (Horizon Discovery Ltd., Cambridge, UK) according to the manufacturer's protocol. Control cells were transfected with ON-TARGETplus Nontargeting Control Pool (Horizon Discovery Ltd., Cambridge, UK). After 48 h, the cells were infected as described above. At 6 h p.i., the cells were collected for protein and RNA isolation. Each experimental condition was tested in triplicate. Silencing efficiency was assessed by western blotting as described above. RNA was extracted using an RNA Blood Mini Kit (QIAGEN, Hilden, Germany) following the manufacturer’s protocols. Real-time PCR analysis of the SARS-CoV-2 N1 gene was carried out as described previously^49,50^.

### Evaluation of SARS-CoV-2 replication over time in breast cancer cell lines

MCF7, MDA-MB-231 and HCC1937 cells were seeded at a density of 7 × 10^5^ cells/well in a 6-well culture plate (Thermo Fisher Scientific, Waltham, MA, USA) in complete RPMI 1640 medium and cultured at 37 °C in a humidified atmosphere containing 5% CO_2_. At 80% confluence, the three breast cancer cell lines were infected with SARS-CoV-2 lineage B.1 as described above. Supernatants and cell pellets were collected at 0, 24, 48, and 72 h and at 7 days post-infection (p.i.). An RNA Blood Mini Kit (QIAGEN, Hilden, Germany) and a NucleoSpin RNA Virus Kit (Macherey Nagel, Duren, Germany) were used to isolate RNA from the pellets and supernatants, respectively, following the manufacturers’ protocols. Real-time PCR analysis of the SARS-CoV-2 N1 gene was carried out as described previously^49,50^.

### MTT assay

MCF7, MDA-MB-231 and HCC1937 cells were seeded at a density of 3000 cells/well in a 96-well culture plate (Thermo Fisher Scientific, Waltham, MA, USA) in complete RPMI 1640 medium and cultured at 37 °C in a humidified atmosphere containing 5% CO_2_. At 80% confluence, the three breast cancer cell lines were infected with SARS-CoV-2 lineage B.1 as described above. Three and 7 days p.i., an MTT assay was performed by adding 20 µL of 3-(4,5-dimethylthiazol-2-yl)-2,5-diphenyltetrazolium bromide (MTT; Sigma‒Aldrich, St. Louis, USA) to each well. The cells were incubated for 3 h at 37 °C in a humidified atmosphere containing 5% CO_2_ in the dark. Then, 100 µL of sodium dodecyl sulfate (SDS) was added. Cell viability was evaluated using a Synergy 4 spectrophotometer (BioTek Instruments, Winooski, USA). Uninfected cells served as controls. Ten replicates were tested for each experimental condition ^50,51^.

### Wound healing assay

MCF7, MDA-MB-231 and HCC1937 cells were seeded at a density of 7 × 10^5^ cells/well in 6-well multiwell culture plates (Thermo Fisher Scientific, Waltham, MA, USA) in complete RPMI 1640 medium and cultured at 37 °C in a humidified atmosphere containing 5% CO_2_. At 100% confluence, the cells were infected with SARS-CoV-2 for 2 h, as described above. After 2 h, the inoculum was removed, and cell-free gaps in the monolayer were generated by mechanical scratching. The cells were then rinsed twice with PBS, and serum-free medium was added. At 0 h, 4 h, and 24 h after mechanical scratching, cell migration into the wound area was evaluated, and the cells were photographed using a Nikon Eclipse Ti Series microscope equipped with a Nikon Digital Sight camera (Nikon, Tokyo, Japan). Uninfected cells served as controls. The unhealed wound area was measured using Photoshop 7.0 software (Adobe, Inc., San Jose, CA, USA). The percentage of gap closure was calculated at each time point by the following formula: 100%-[(unhealed area at a specific time-point/area at 0 h) × 100].

### Colony formation assay

MCF7, MDA-MB-231 and HCC1937 cells were seeded at a density of 500 cells/well in 6-well plates (Thermo Fisher Scientific, Waltham, MA, USA) in complete RPMI 1640 medium and cultured at 37 °C in a humidified atmosphere containing 5% CO_2_. After 24 h, the cells were infected with SARS-CoV-2 as described above. After 14 days, the colonies were fixed with 3.7% paraformaldehyde and stained with a solution of 0.5% crystal violet (Merck KGaA, Darmstadt, Germany) in 20% ethanol in H_2_O. The wells were then washed with H_2_O and subsequently dried at room temperature. Each experimental condition was tested in triplicate. Colonies were photographed using the Essential V6 Imaging Platform (Uvitec Cambridge, Cambridge, UK), and the images were analyzed with ImageJ software. The data are expressed as the percentage of the total area of each well that was positive for crystal violet staining.

### Immunofluorescence analysis

MCF7, MDA-MB-231 and HCC1937 cells (50,000 cells/well) were plated on glass coverslips (Thermo Fisher Scientific, Waltham, MA, USA) in 12-well plates and grown overnight. The following day, the cells on the coverslips were washed with 1X PBS, fixed with 3.7% formalin for 20 min, washed with PBS and permeabilized with a solution of 0.1% Triton X-100 (Merck KGaA, Darmstadt, Germany) in PBS for 10 min on ice. Nonspecific binding site saturation was performed at room temperature for 1 h with PBS containing 3% bovine serum albumin (BSA; Merck KGaA, Darmstadt, Germany), and the coverslips were then incubated with a recombinant rabbit monoclonal anti-ACE2 antibody (clone SN0754; Thermo Fisher Scientific, Waltham, MA, USA) diluted 1:100 in 0.01% Triton X-100/1% BSA in PBS for 1 h at room temperature. The coverslips were then incubated with an Alexa Fluor 488-conjugated goat anti-rabbit antibody (Immunological Sciences, Rome, Italy) diluted 1:500 in 0.01% Triton X-100/1% BSA solution in PBS for 1 h at room temperature. Nuclei were counterstained with 4′,6-diamidino-2-phenylindole dihydrochloride (DAPI; Thermo Fisher Scientific Inc., Waltham, MA, USA) for 10 min at room temperature. The coverslips were mounted on glass slides using ProLong™ Gold Antifade Mountant (Thermo Fisher Scientific Inc., Waltham, MA, USA), and staining was analyzed using a Leica TCS SP8 X laser scanning confocal microscope (Leica Microsystems GmbH, Mannheim, Germany).

### Gene expression profiling

Gene expression profiling was performed at Fondazione IRCCS Istituto Nazionale dei Tumori di Milano (Milan, Italy), as described previously^52^. MCF7, MD AMB231 and HCC1937 cells were seeded in quadruplicate at a density of 7 × 10^5^ cells/well in a 6-well culture plate (Thermo Fisher Scientific, Waltham, MA, USA) in complete RPMI 1640 medium and cultured at 37 °C in a humidified atmosphere containing 5% CO_2_. At 80% confluence, the three breast cancer cell lines were infected with SARS-CoV-2 lineage B.1 as described above. At twenty-four hours and at 7 days p.i., total RNA was extracted with QIAzol Lysis Reagent (QIAGEN, Hilden, Germany) and an RNeasy Mini Kit (QIAGEN, Hilden, Germany) according to the manufacturer’s instructions. A 4200 TapeStation instrument (Agilent Technologies, Santa Clara, CA, USA) and a Qubit fluorometer (Thermo Fisher Scientific, Waltham, MA, USA) were utilized for RNA quality assessment and quantification, respectively. Gene profiling was performed using the Clariom™ S Assay, human (Thermo Fisher Scientific, Waltham, MA, USA), following the manufacturer’s protocols.

### Bioinformatic analysis

CEL files generated during the Clariom™ S Assay were imported into Transcriptomic Analysis Console (TAC; version 4.0.2.15, Thermo Fisher Scientific, Waltham, MA, USA), and the raw data were log2-transformed and normalized using the robust multichip average (RMA) algorithm. Normalized data were collapsed from the probe to the gene level using the collapseRows function of the WGCNA R package^53^. For each gene, the probe with the highest variation across samples was selected^54^. Differentially expressed genes (DEGs) between SARS-CoV-2-infected and control breast cancer cells were identified using the limma R package with the log2-normalized data^55^. *p* Values were corrected for multiple testing using the Benjamini‒Hochberg false discovery rate (FDR) procedure, and DEGs with an FDR < 0.05 were considered statistically significant. An online tool (https://bioinformatics.psb.ugent.be/webtools/Venn) was used to generate Venn diagrams to identify overlapping up- and downregulated DEGs in the three breast cancer cell models. Functional enrichment analysis was carried out by preranked gene set enrichment analysis (GSEAPreranked) using the javaGSEA Desktop Application v4.2.3.0^56^ with canonical pathway gene sets derived from the Reactome pathway database, which contains 1654 gene sets (c2.cp.reactome.v2022.1.Hs.symbols). Genes were ranked according to the t test value determined via analysis with the limma package. Gene sets with an FDR q value < 0.05 were considered statistically significant. Metascape analysis was carried out through a web-based platform (https://Metascape.org/gp/index.html#/main/step1) using default parameters^57^. The “SARS-CoV-2 Metagene” was generated by averaging the log2-transformed normalized expression values of the 23 common genes upregulated by SARS-CoV-2 infection, as determined by microarray analysis, in all three breast cancer cell lines 24 h p.i. in the Molecular Taxonomy of Breast Cancer International Consortium (METABRIC) dataset (https://ega-archive.org/). Twenty-one of the 23 genes constituting the SARS-CoV-2 Metagene were included in the METABRIC dataset. The metagene score was used to define two breast cancer patient groups, named the “high” and “low” SARS-CoV-2 Metagene score groups, using the lower tertile as the threshold. The microarray data and sample information have been deposited in the Gene Expression Omnibus database (https://www.ncbi.nlm.nih.gov/gds) under accession numbers GSE234242 (gene profiling of breast cancer cell lines 24 h p.i.) and GSE234236 (gene profiling of breast cancer cell lines 7 days p.i.).

### In vitro studies with Tamoxifen

MCF7 cells were seeded at a density of 5 × 10^5^ cells/well in a 6-well culture plate (Thermo Fisher Scientific, Waltham, MA, USA) in complete RPMI 1640 medium and cultured at 37 °C in a humidified atmosphere containing 5% CO_2_. The following day, the cells were treated with 10 µM Tamoxifen. After 24 h, mRNA was extracted using Direct-zol™ RNA MicroPrep (Zymo Research, Irvine, CA, USA) and reverse-transcribed with a High-Capacity RNA-to-cDNA Kit (Applied Biosystems, Thermo Fisher Scientific, Waltham, MA, USA) according to the manufacturer’s instructions. Real-time PCR was performed as described in^58^ using the following TaqMan® gene expression assays (Applied Biosystems, Thermo Fisher Scientific, Waltham, MA, USA): *BCL2* (Hs04986394_s1), *PGR* (Hs01556702_m1) and Glyceraldehyde-3-Phosphate Dehydrogenase (GAPDH, Hs02786624_g1). The mRNA level was normalized to that of GAPDH. The expression data were analyzed by the 2^-ΔCt^ method^58,59^.

MCF7 cells were seeded in 96-well plates (3 × 10^4^ cells/well) in complete medium and allowed to adhere overnight at 37 °C with 5% CO_2_ to 80% confluence. Cell infection with SARS-CoV-2 lineage B.1 was performed as described above. Tamoxifen at a final concentration of 10 µM was added to the MCF7 cells at different time points: 24 h before SARS-CoV-2 infection, at the time of infection and immediately after infection. Moreover, an additional group in which Tamoxifen was present throughout the experiment was included. Untreated infected cells served as a control for viral replication. Viral RNA was extracted from the cell supernatants at 24, 48, 72 h and at 7 days p.i. and was subjected to real-time PCR analysis as described above.

At 7 days p.i., 250 ng of RNA obtained from infected MCF7 cells untreated or exposed to Tamoxifen throughout the experiment was reverse transcribed using a QuantiTect Reverse Transcription Kit (QIAGEN, Hilden, Germany) according to the manufacturer’s instructions. The expression levels of *OAS1*, *IFIT1*, *IFIT3*, and *IFI6* were measured by real-time PCR with SYBR Green QuantiTect (QIAGEN, Hilden, Germany) using the following primers: *OAS1* (Forward: AGGAAAGGTGCTTCCGAGGTAG, Reverse: GGACTGAGGAAGACAACCAGGT), *IFIT1* (Forward: GCCTTGCTGAAGTGTGGAGGAA, Reverse: ATCCAGGCGATAGGCAGAGATC), *IFIT3* (Forward: CCTGGAATGCTTACGGCAAGCT, Reverse: GAGCATCTGAGAGTCTGCCCAA), *IFI6* (Forward: TGATGAGCTGGTCTGCGATCCT, Reverse: GTAGCCCATCAGGGCACCAATA), and *GAPDH* (Forward: GCCCAGGATGCCCTTGA, Reverse: GTGTCCCCACTGCCAAC). Amplification was carried out using a 7500 Real Time PCR System (Thermo Fisher, Waltham, MA, USA). Each mRNA level was normalized to that of GAPDH. The data were analyzed by the 2^-ΔCt^ method.

### WST assay

MCF7 cells were seeded at a density of 2500 cells/well in a 96-well culture plate (Thermo Fisher Scientific, Waltham, MA, USA) in complete RPMI 1640 medium and cultured at 37 °C in a humidified atmosphere containing 5% CO_2_. The following day, the cells were exposed to 10 µM Tamoxifen under the experimental conditions described in the above section. Eight replicates were tested for each experimental condition. After 7 days, cell proliferation was evaluated with CytoSelect™ WST-1 Cell Proliferation Assay Reagent (Cell Biolabs Inc., San Diego, CA, USA) following the manufacturer’s protocols.

### Senescence-associated β-galactosidase (SA-β-Gal) staining

MCF7 cells were seeded at 2 × 10^5^ cells/well in 24-well multiwell culture plates (Thermo Fisher Scientific, Waltham, MA, USA) in complete RPMI 1640 medium and cultured at 37 °C in a humidified atmosphere containing 5% CO_2_. At 80% confluence, a final concentration of 10 µM Tamoxifen was added to the MCF7 cells either 24 h before SARS-CoV-2 infection, during infection, or immediately after infection. The cells were infected as described above. Infected cells not treated with Tamoxifen served as controls. At 7 days p.i., SA-β-Gal staining was carried out using a β-Gal Staining Kit (Invitrogen, Thermo Fisher Scientific, Waltham, MA, USA) following the manufacturer's instructions. Each experimental condition was tested in quadruplicate. The samples were observed via phase contrast microscopy, and SA-β-Gal quantification was performed with ImageJ 1.53e software (ImageJ, NIH, USA) ^60^. Briefly, images were converted to grayscale, and the threshold parameters were adjusted to evaluate only the SA-β-Gal-positive area, which was then expressed as a percentage of the total image area.

### Plaque reduction assay

The viral titer in supernatants from treated and untreated infected MCF7 cells was assessed by means of a plaque reduction assay. A total of 4 × 10^5^ VERO E6 cells/well were seeded in a 6-well plate in complete medium and allowed to adhere overnight at 37 °C in 5% CO_2_ to 80% confluence. Five hundred microliters of tenfold serial dilutions of the supernatant collected at 7 days p.i. was used to infect VERO E6 cells for 2 h in duplicate. After removal of the viral inoculum, complete medium with 3% agarose was added to the cells, which were then cultured for 48 h at 37 °C with 5% CO_2_. Then, the cells were fixed with 3.7% paraformaldehyde and stained with methylene blue. The viral titer was calculated as plaque-forming units (PFU) per milliliter (PFU/mL).

### Statistical analysis

GraphPad Prism version 10.2.2 (GraphPad Software, San Diego, CA, USA) was used for statistical analysis. Normality tests were performed prior to each statistical analysis to ascertain the normality of the data distribution. Differences between two groups were determined by two-tailed unpaired Student’s t test or by the Mann–Whitney U test for nonparametric data. Comparisons among 3 or more experimental groups were performed by one-way ANOVA followed by Tukey’s multiple comparison test with a single pooled variance or by the Kruskal–Wallis test followed by Dunn’s multiple comparison test for nonparametric data. The data are presented as the means ± standard errors of the mean (means ± SEMs). Pearson correlation coefficient (r) and the corresponding *p*-value between gene expressions and viral load was computed overall. The resulting scatter plot was visually inspected considering the stratification by cell lines. The Kaplan–Meier method was utilized to perform overall survival analysis. Univariate and multivariate survival analyses were carried out with Cox proportional hazards regression models with IBM SPSS Statistics Standard version 28 (SPSS, Chicago, IL, USA). All predictors with a *p* value < 0.1 in univariate analysis were utilized for subsequent multivariate analysis. Hazard ratios (HRs) and the corresponding 95% confidence intervals (CIs) were used to quantify the effects of the explanatory variables on event hazards. Differences were considered significant at *p* < 0.05.

### Supplementary Information

Supplementary Information 1.

Supplementary Information 2.

Supplementary Information 3.

Supplementary Information 4.

Supplementary Information 5.

Supplementary Information 6.

Supplementary Information 7.

Supplementary Information 8.

## Data Availability

The datasets generated during the current study are available in the Gene Expression Omnibus (GEO) repository (https://www.ncbi.nlm.nih.gov/gds) under the accession numbers GSE234242 and GSE234236.
