# Supplementary material for: Impact of in vitro SARS-CoV-2 infection on breast cancer cells
Source: Sci Rep. 2024 Jun 7;14:13134. doi: 10.1038/s41598-024-63804-3 (PMC11161491; doi:10.1038/s41598-024-63804-3)
Supplement: Supplementary file 1 — Supplementary Information 1. [file 41598_2024_63804_MOESM1_ESM.pdf]

Impact of *in vitro* SARS-CoV-2 infection on breast cancer cells

Michele Sommariva[1,2], Maria Dolci[3], Tiziana Triulzi[2], Federico Ambrogi[4], Matteo Dugo[5], Loris De Cecco[6], Valentino Le Noci[1], Giancarla Bernardo[1], Martina Anselmi[1], Serenella Pupa[2], Lucia Signorini[3], Nicoletta Gagliano[1], Lucia Sfondrini[1,2], Serena Delbue[3], Elda Tagliabue[2]

[1]Dipartimento di Scienze Biomediche per la Salute, Università degli Studi di Milano, via Mangiagalli 31, 20133 Milan (Italy)

[2]Microambiente e Biomarcatori dei Tumori Solidi, Dipartimento di Oncologia Sperimentale, Fondazione IRCCS Istituto Nazionale dei Tumori di Milano, via Amadeo 42, 20133 Milan (Italy)

[3]Dipartimento di Scienze Biomediche, Chirurgiche ed Odontoiatriche, Università degli Studi di Milano, via Pascal 36, 20133 Milan (Italy)

[4]Dipartimento di Scienze Cliniche e di Comunita, Università degli Studi di Milano, Via Celoria 22, 20133 Milan (Italy)

[5]Department of Medical Oncology, IRCCS Ospedale San Raffaele, Via Olgettina 60, 20132 Milan (Italy)

[6]Integrated Biology of Rare Tumors, Dipartimento di Oncologia Sperimentale, Fondazione IRCCS Istituto Nazionale dei Tumori di Milano, via Amadeo 42, 20133 Milan (Italy)

Supplementary Figure S1

a b c

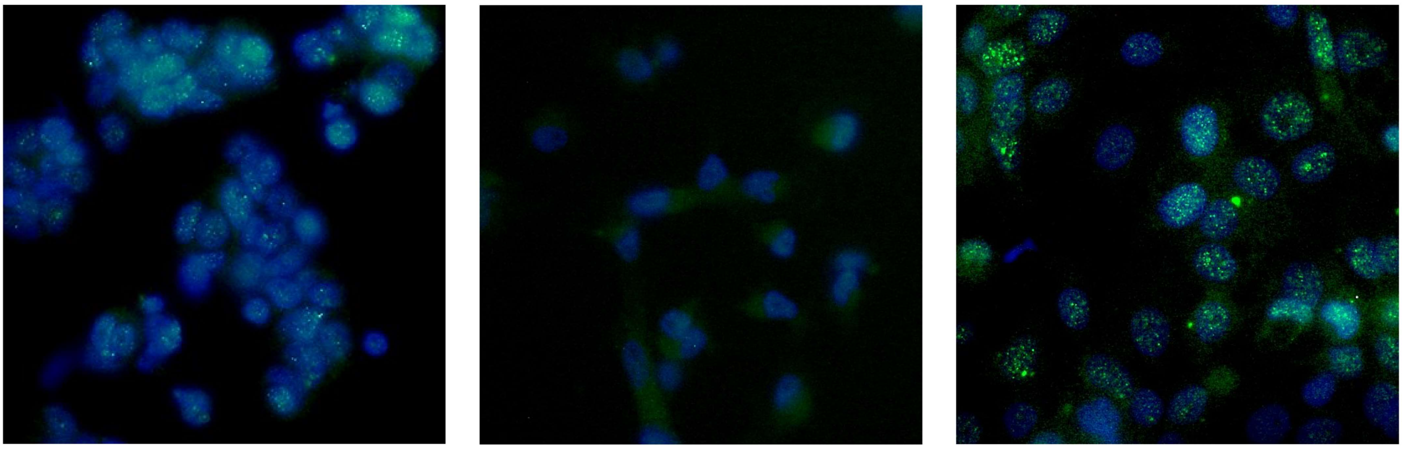

Supplementary Figure S1. Evaluation of ACE2 expression in breast cancer cell lines by immunofluorescence analysis

Representative immunofluorescence image of SARS-CoV-2 receptor ACE2 protein expression (green signals) in the MCF7 (a), MDA-MB-231 (b) and HCC1937 (c) cell lines, as determined by immunofluorescence analysis. Nuclei were counterstained with 4′,6-diamidino-2-phenylindole (DAPI; blue). Magnification, 60x.

Supplementary Figure S2

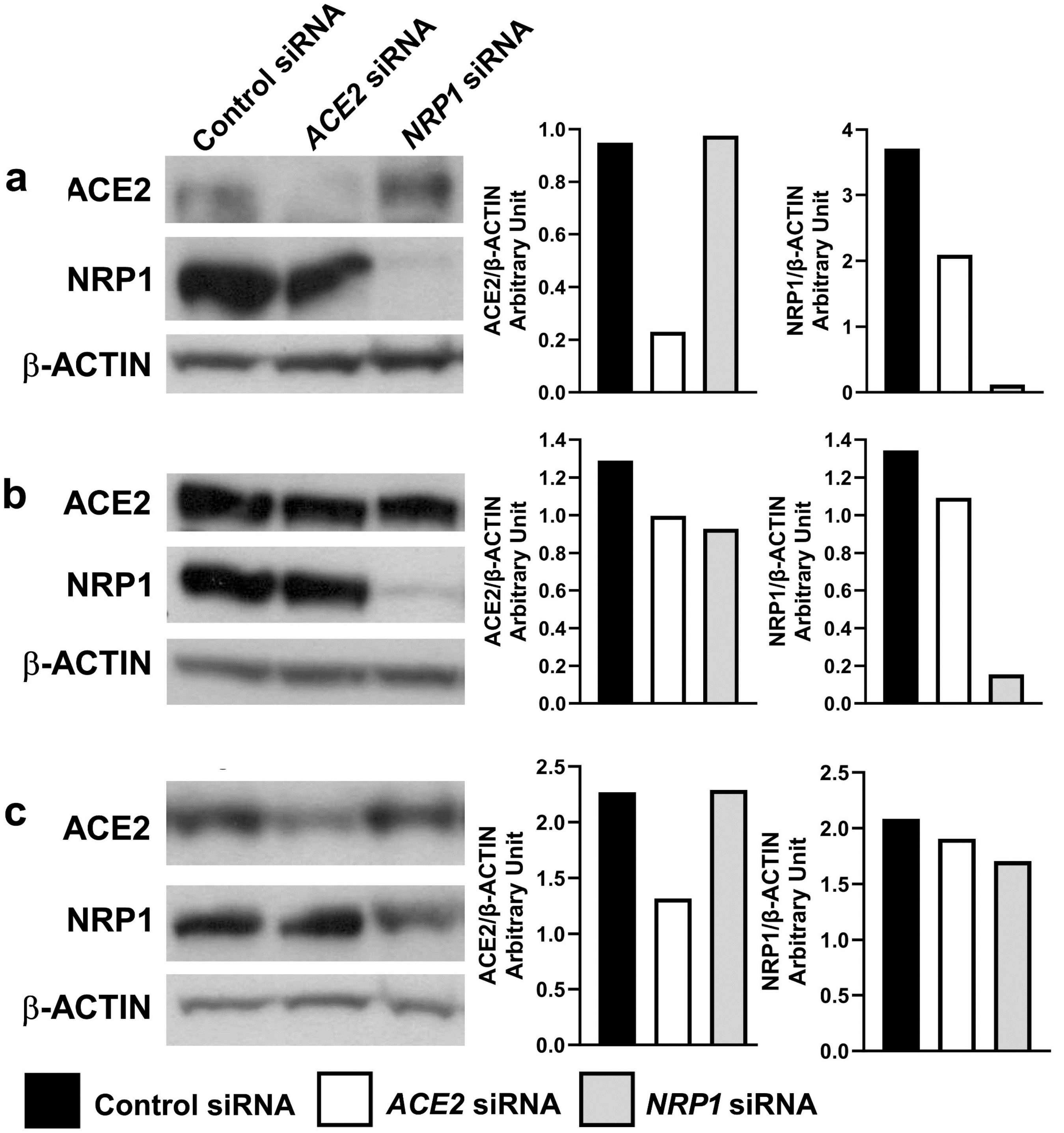

Supplementary Figure S2. ACE2 and NRP1 silencing in breast cancer cell lines

ACE2 and NRP1 were silenced in the MCF7, MDA-MB-231 and HCC1937 cell lines using specific siRNAs. Forty-eight hours after silencing, the cells were infected with SARS-CoV-2, as detailed in the Methods section. After 6 hours, the cells were collected, and proteins were isolated. Protein expression was evaluated by Western blotting and densitometric analysis. GAPDH was used to normalize protein loading per lane.

Supplementary Figure S3

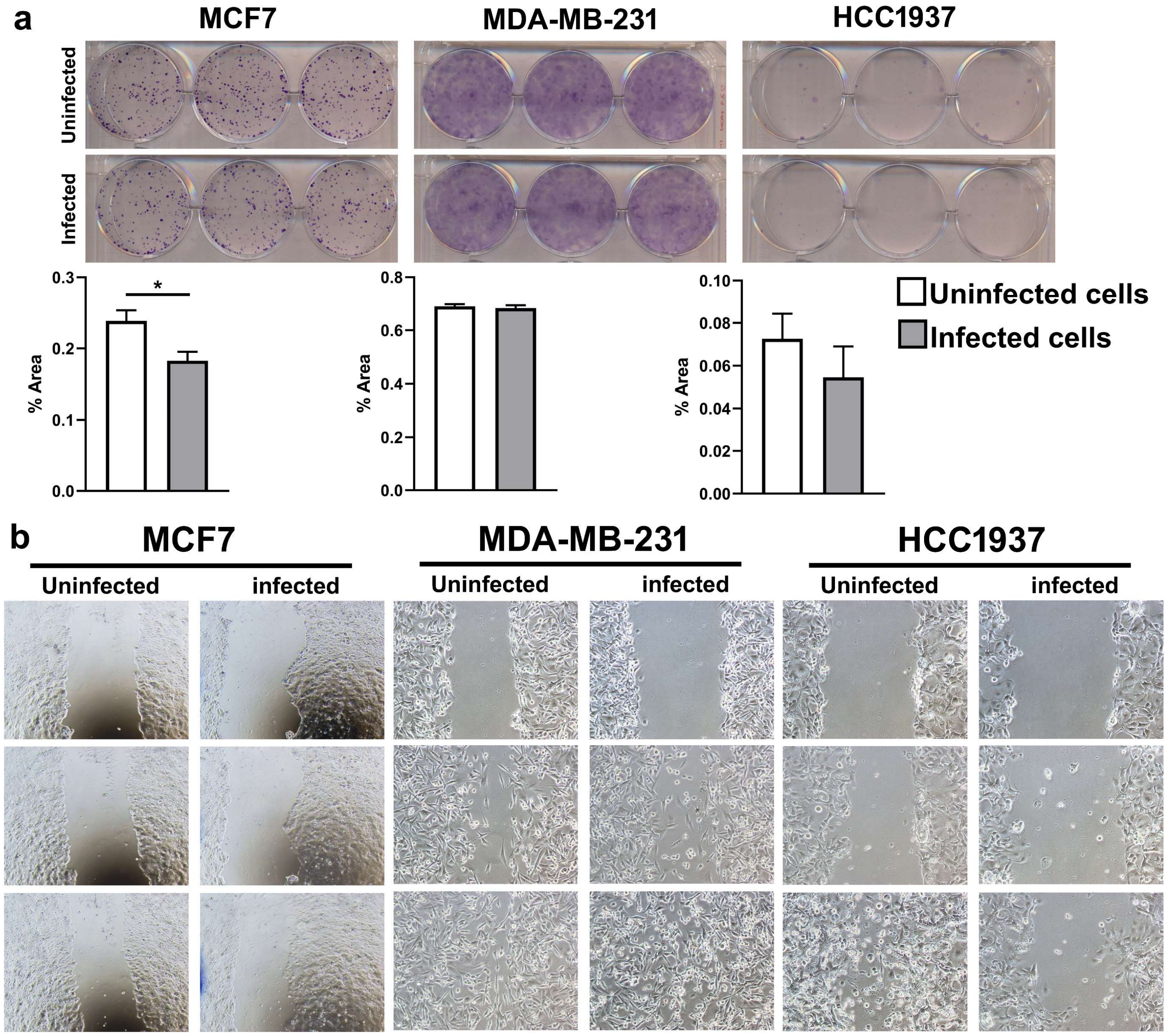

Supplementary Figure S3. Effect of SARS-CoV-2 infection on breast cancer cell proliferation and migration

(**a**) MCF7, MDA-MB-231 and HCC1937 cells were seeded at a density of 500 cells/well. The following day, the cells were infected with SARS-CoV-2, and after 14 days, the colonies were stained with crystal violet. Uninfected cells served as controls. The data are presented as the mean ± SEM of the percentage of crystal violet-positive area, as evaluated by ImageJ software, and are representative of one of three independent experiments with similar results. *$p < 0.05$ by two-tailed unpaired Student’s t test. (**b**) Representative images of cell migration in wounded MCF-7, MDA-MB-231 and HCC1937 cell monolayers with or without SARS-CoV-2 infection. Magnification, 4x.

Supplementary Figure S4

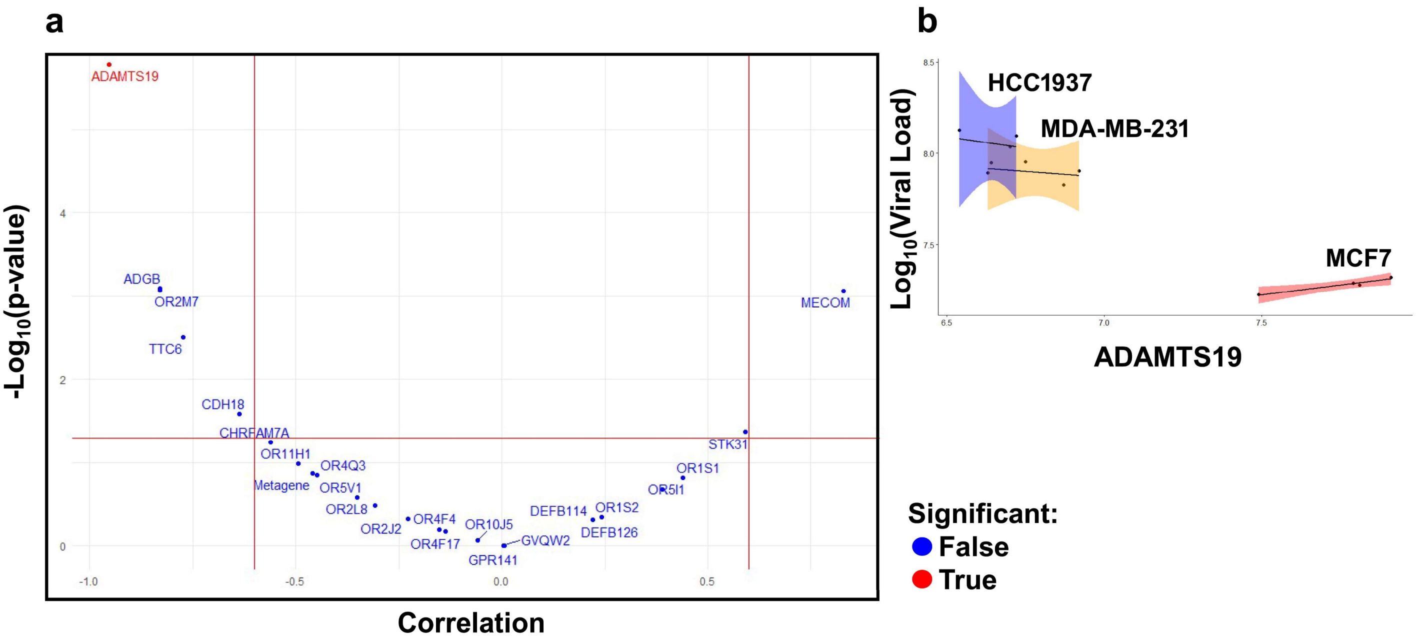

Supplementary Figure S4. Correlation between the 23 overlapping genes upregulated by SARS-CoV-2 infection in the three cancer cell lines and the viral load

(**a**) Correlation analysis of the expression levels of the 23 overlapping genes upregulated 24 hours post-infection in all three infected cell lines compared to their uninfected counterparts with the viral load. The volcano plot shows the viral load on the x-axis and the p value, corrected for multiple comparisons, on the y-axis. Both values are presented on a log10 scale. (**b**) Correlation analysis of the ADAMTS19 gene expression level and the viral load in each of the three breast cancer cell lines.

Supplementary Figure S5

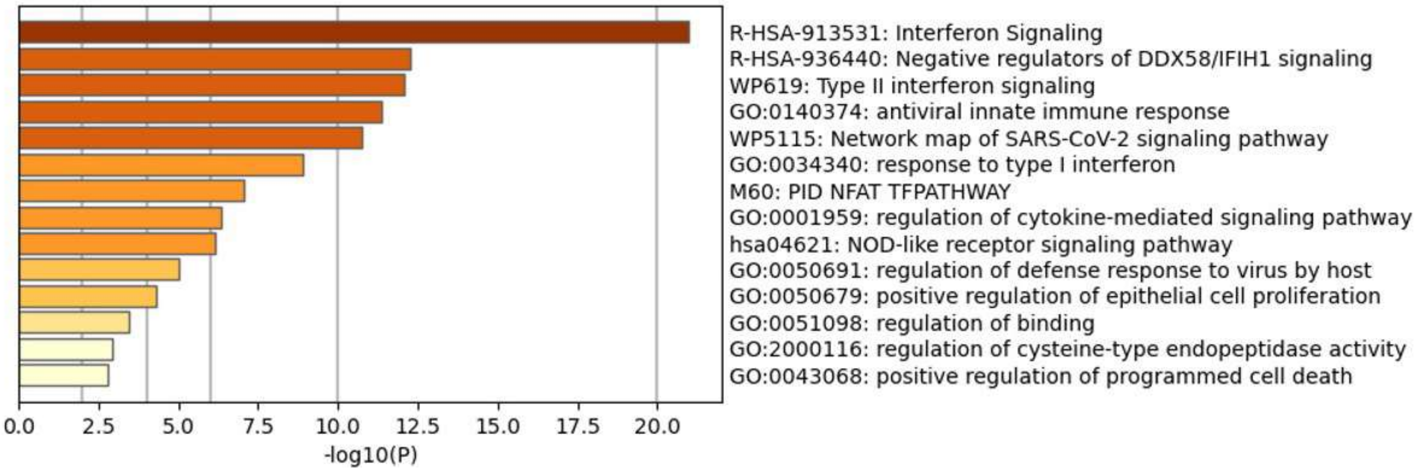

Supplementary Figure S5. Metascape analysis of the 28 upregulated DEGs in infected MCF7 cells at 7 days p.i.

List of 14 clusters identified by Metascape functional enrichment analysis of the 28 upregulated DEGs in the infected MCF7 cell line at 7 days p.i. Each cluster is colored according to the p value. Log10 (p) is the log10-transformed p value.

Supplementary Figure S6

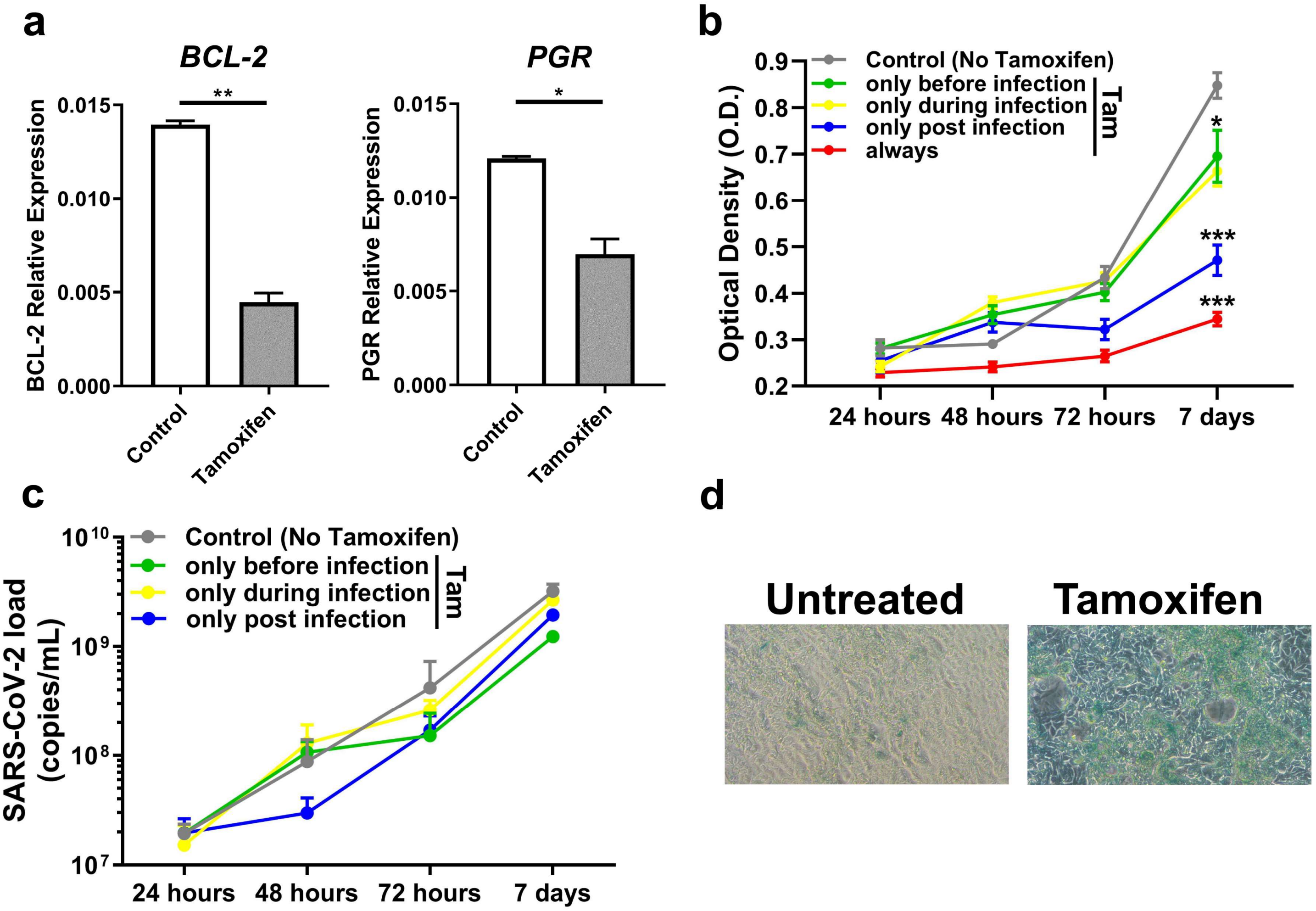

Supplementary Figure S6. Effect of Tamoxifen on MCF7 cells

(**a**) Real-time PCR analysis of *BCL-2* and *PGR* gene expression in MCF7 cells treated with 10 μM Tamoxifen for 24 hours. The control cells were left untreated. The expression levels were normalized to that of *GAPDH* as the housekeeping reference gene, and relative expression was analyzed by the $2^{-\Delta Ct}$ method. (**b**) MCF7 cells were exposed to 10 μM tamoxifen 24 hours before infection (only before infection), at the time of infection (only during infection), immediately post infection (only post infection) and throughout the experiment (always). Cell viability was assessed by a WST-1 cell proliferation assay at 7 days p.i. Control cells were left untreated. O.D.: optical density. Tam: Tamoxifen. The data are presented as the means ± SEMs and are representative of one of three independent experiments with similar results. * $p < 0.05$, ** $p < 0.01$ ***$p < 0.001$ versus untreated control cells at 7 days p.i. by one-way ANOVA followed by Tukey's multiple comparison test with a single pooled variance. (**c**) SARS-CoV-2-infected MCF7 cells were exposed to 10 μM tamoxifen 24 hours before infection (only before infection), at the time of infection (only during infection) and immediately post infection (only post infection). The viral load in the supernatants was evaluated at 24, 48, and 72 hours and at 7 days p.i. by real-time PCR. The data, expressed as copies/mL, are presented as the means ± SEMs and are representative of one of three independent experiments with similar results. (**d**) Representative images of SA-β-Gal staining 7 days after infection of MCF7 cells exposed to 10 μM Tamoxifen throughout the experiment or left untreated. Magnification, 20x.

Supplementary Figure S7

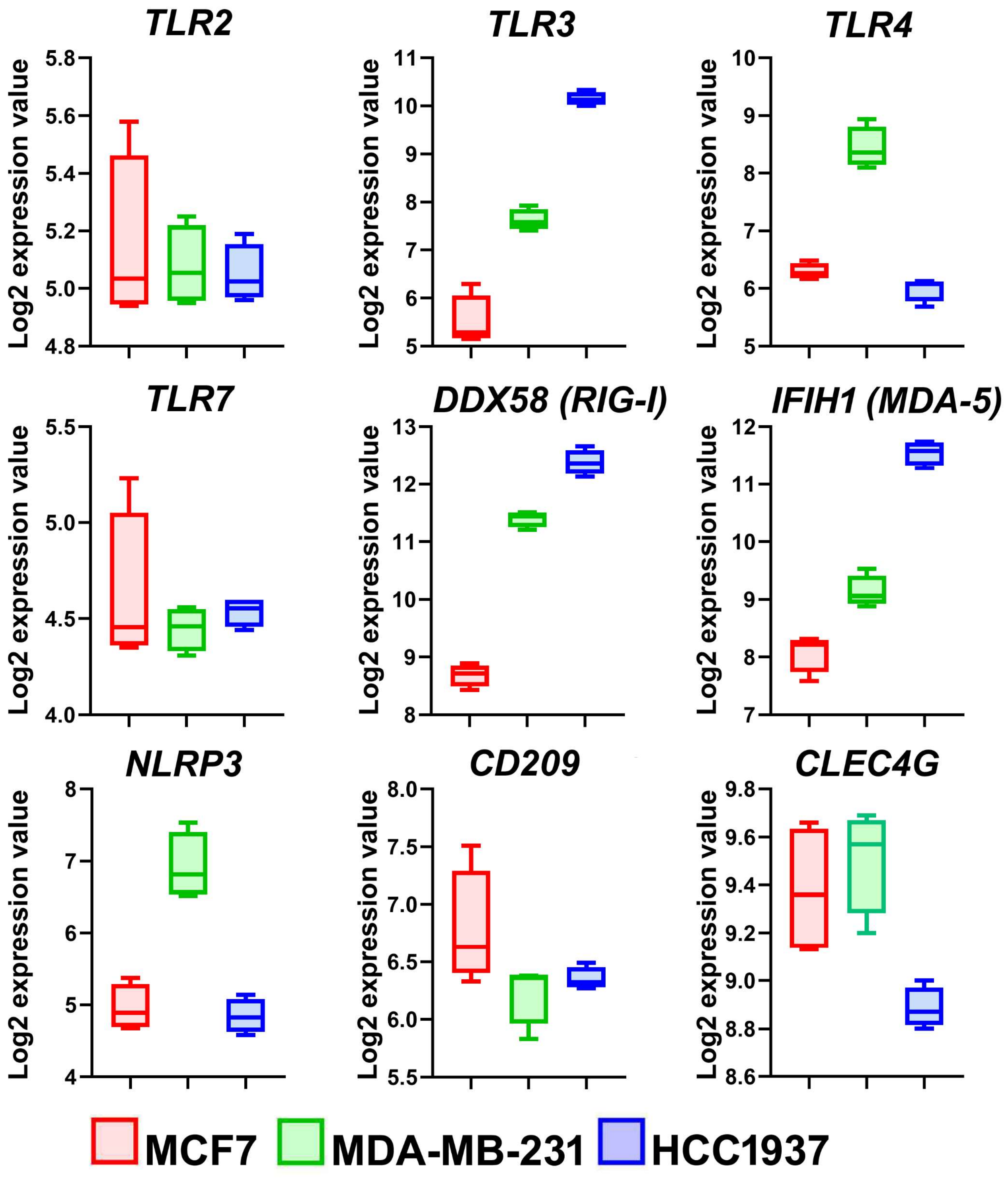

Supplementary Figure S7. Expression of viral recognition immune receptors in the 3 breast cancer cell lines

Log2-transformed and RMA-normalized microarray expression data for innate immune receptors retrieved from the gene expression profiles of uninfected cells of the three breast cancer cell lines (24 hours post-infection).
