# Supplementary material for: Impact of in vitro SARS-CoV-2 infection on breast cancer cells
Source: Sci Rep. 2024 Jun 7;14:13134. doi: 10.1038/s41598-024-63804-3 (PMC11161491; doi:10.1038/s41598-024-63804-3)
Supplement: Supplementary file 2 — Supplementary Information 2. [file 41598_2024_63804_MOESM2_ESM.docx]

**SUPPLEMENTARY TABLE LEGENDS**

**Supplementary Table S1. DEGs between infected and uninfected breast cancer cells 24 hours after SARS-CoV-2 infection**

MCF7, MDA-MB-231 and HCC1937 breast cancer cell lines were infected with SARS-CoV-2 lineage B1 and gene expression profile analysis was performed 24 hours p.i. DEGs between SARS-CoV-2-infected and control breast cancer cells were determined using limma R package on log2 normalized data and p values were corrected for multiple testing using the Benjamini-Hochberg false discovery rate (FDR) method. DEGs were considered statistically significant at a FDR < 0.05.

**Supplementary Table S2. 23 common up-modulated DEGs in the three breast cancer cell lines 24 hours after SARS-CoV-2 infection**

Lists of the 23 DEGs resulted commonly up-modulated in infected breast in comparison to control cells 24 hours p.i.

**Supplementary Table S3. DEGs between infected and uninfected breast cancer cell lines 7 days after SARS-CoV-2 infection**

MCF7, MDA-MB-231 and HCC1937 breast cancer cell lines were infected with SARS-CoV-2 lineage B1 and gene expression profile analysis was performed 7 days p.i. DEGs between SARS-CoV-2-infected and control breast cancer cells were determined using limma R package on log2 normalized data and p values were corrected for multiple testing using the Benjamini-Hochberg false discovery rate (FDR) method. DEGs were considered statistically significant at a FDR < 0.05.

**Supplementary Table S4. List of statistically significant enriched pathways in infected breast cancer cell lines by GSEA analysis**

MCF7, MDA-MB-231 and HCC1937 breast cancer cell lines were infected with SARS-CoV-2 lineage B1 and gene expression profile analysis was performed 24 hours and 7 days p.i. The table lists the statistically significant pathways (FDR q-value < 0.05) found enriched in infected breast cancer cell lines compared to control cells by preranked GSEA analysis.

**Supplementary Table S5. Clinical characteristics of Luminal A breast cancer patients utilized for survival analysis**

Clinicopathological characteristics of Luminal A breast cancer patients present in the METABRIC dataset utilized for survival analysis. P value was determined by *Chi-square and §Fisher's exact test.
