# Supplementary material for: Impact of in vitro SARS-CoV-2 infection on breast cancer cells
Source: Sci Rep. 2024 Jun 7;14:13134. doi: 10.1038/s41598-024-63804-3 (PMC11161491; doi:10.1038/s41598-024-63804-3)
Supplement: Supplementary file 7 — Supplementary Information 7. [file 41598_2024_63804_MOESM7_ESM.docx]

**Supplementary Table 4. Clinical characteristics of Luminal A breast cancer patients present in the METABRIC dataset and utilized for survival analysis.**

| **Variable** | **Total** | **SARS-Cov-2 Metagene score** | | **p-value** |
| --- | --- | --- | --- | --- |
|  |  | **Low** | **High** |  |
| **SARS-CoV-2 Metagene** | 679 | 224 | 455 |  |
| **Lymph Node Metastasis** |  |  |  |  |
| Negative | 392 | 125 | 267 | 0.5089^§^ |
| Positive | 267 | 99 | 188 |  |
| **Tumor Grade** |  |  |  |  |
| G1 | 117 | 44 | 73 | 0.6691^*^ |
| G2 | 363 | 114 | 249 |  |
| G3 | 168 | 56 | 112 |  |
| NA | 31 | 10 | 21 |  |
| **Tumor Size** |  |  |  |  |
| T1 | 339 | 114 | 225 | 0.1065^*^ |
| T2 | 320 | 108 | 212 |  |
| T3 | 19 | 2 | 17 |  |
| NA | 1 | 0 | 1 |  |
| **Hormone Therapy** |  |  |  |  |
| No | 218 | 64 | 154 | 0.1898^§^ |
| Yes | 461 | 160 | 301 |  |
| **Inferred menopausal state** |  |  |  |  |
| Pre | 28 | 6 | 22 | 0.0023^§^ |
| Post | 336 | 98 | 238 |  |

P value was determined by **^*^**Chi-square and **^§^**Fisher's exact test
