# Supplementary material for: Impact of in vitro SARS-CoV-2 infection on breast cancer cells
Source: Sci Rep. 2024 Jun 7;14:13134. doi: 10.1038/s41598-024-63804-3 (PMC11161491; doi:10.1038/s41598-024-63804-3)
Supplement: Supplementary file 8 — Supplementary Information 8. [file 41598_2024_63804_MOESM8_ESM.pdf]

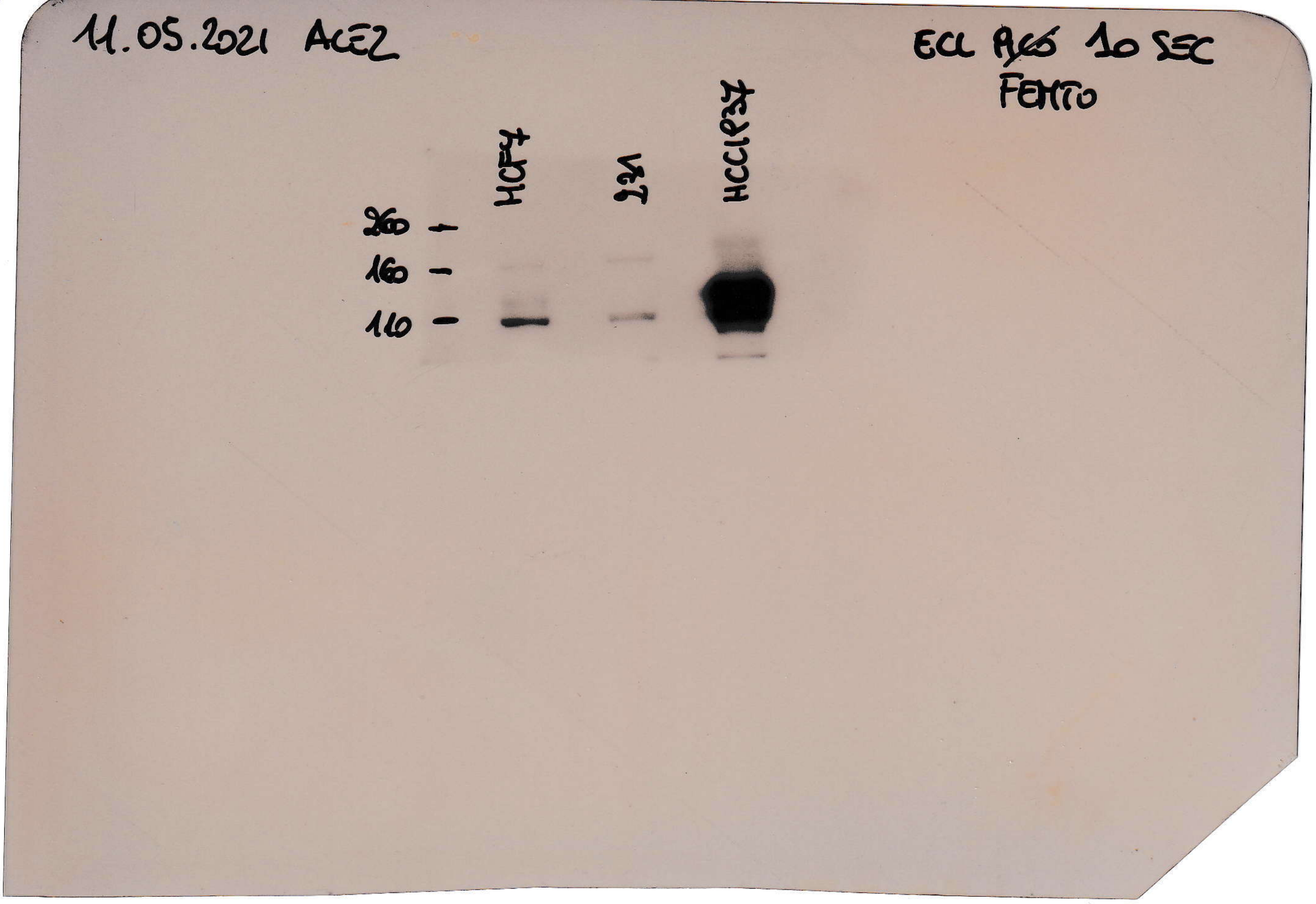
11.05.2021 ACE2
ECL ~~Pico~~ 10 SEC
FEMTO
MCF7
293
HCC1937
260
160
110

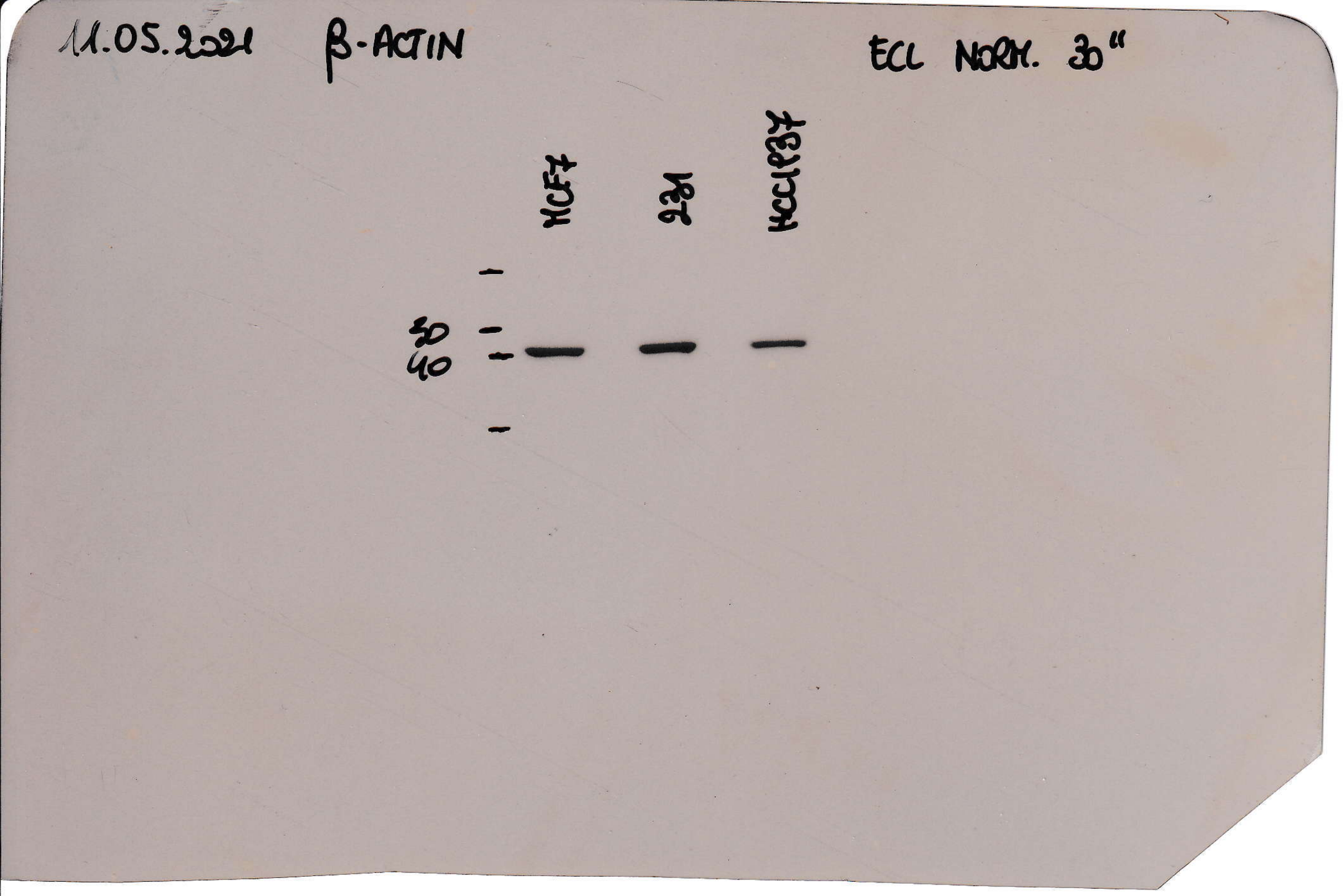

11.05.2021
β-ACTIN
ECL NORM. 30"
MCF7
231
HCC1937
50
40

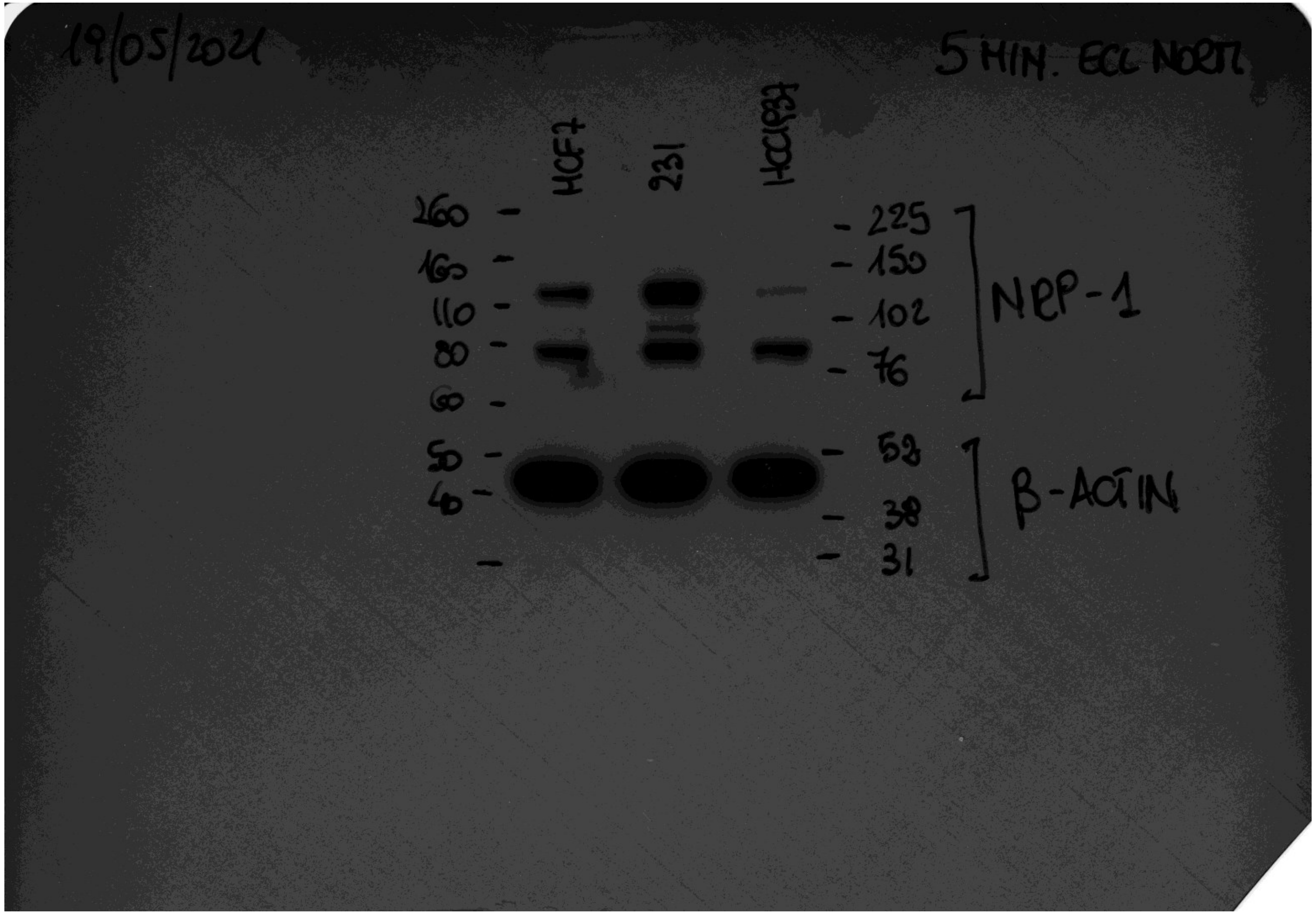
19/05/2021
5 MIN. ECL NORM
MCF7
231
260
160
110
80
60
50
40
225
150
102
76
52
38
31
NRP-1
β-ACTIN

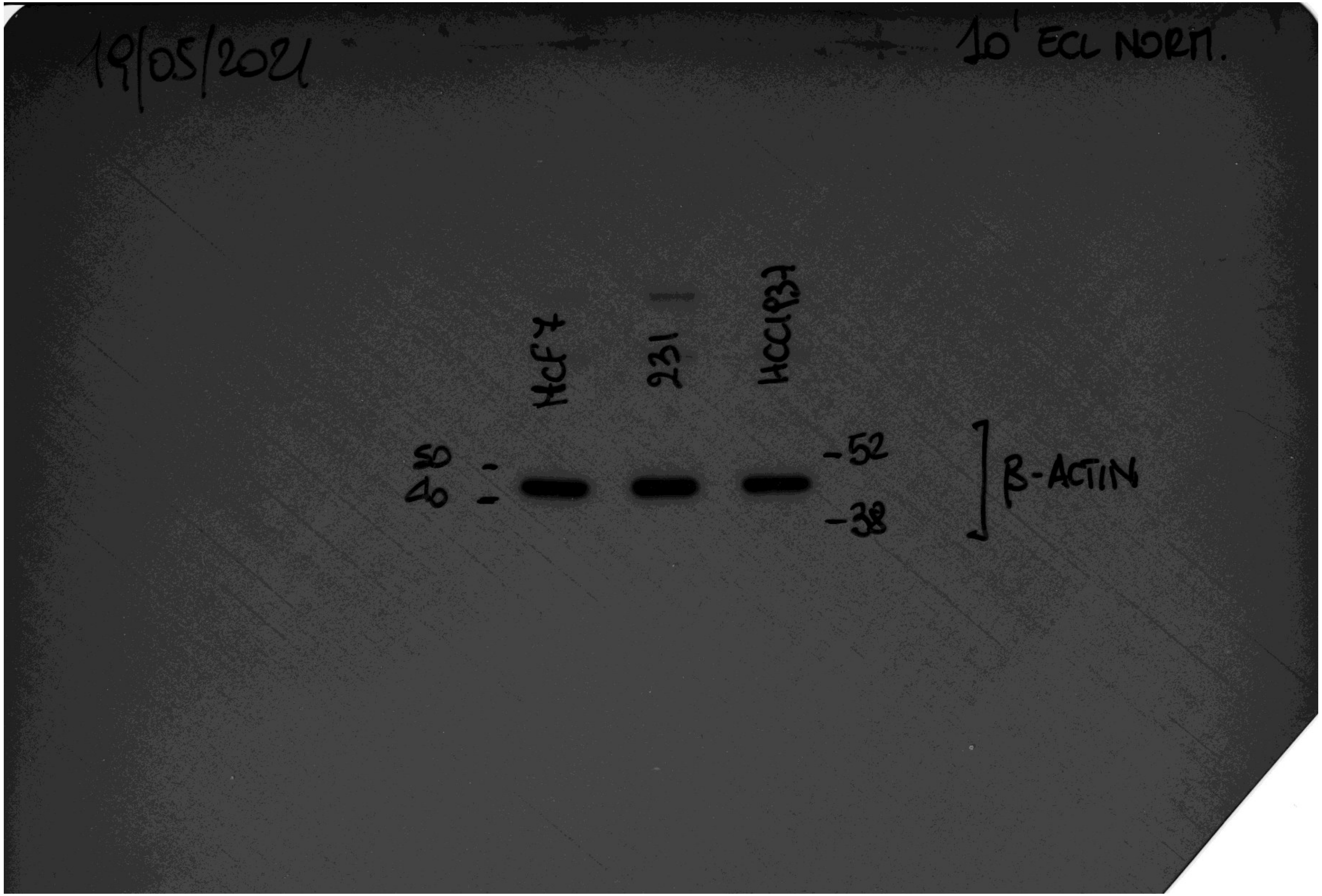
19/05/2021
10' ECL NORM.
MCF7
231
HCC1937
50
40
52
38
β-ACTIN

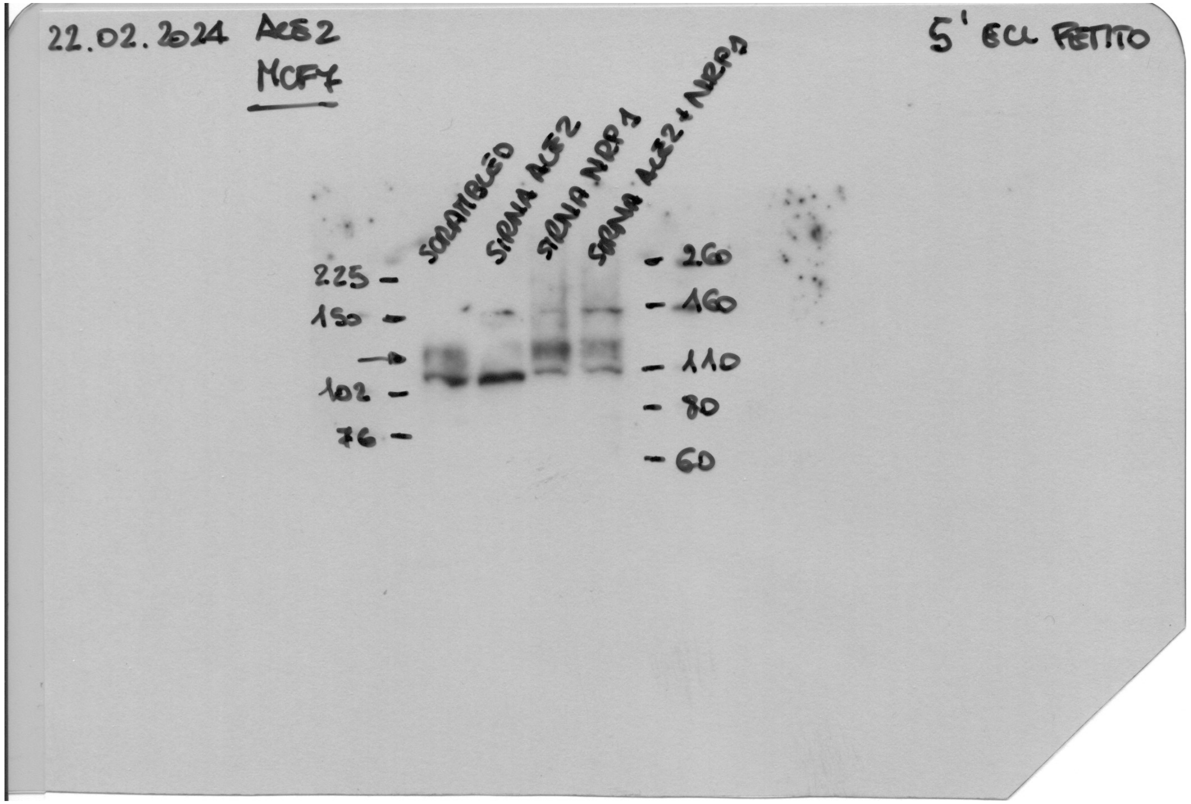
22.02.2024
ACE2
MCF7
5' ECL FETITO
SCRAMBLED
SIRNA ACE2
SIRNA NRP1
SIRNA ACE2 + NRP1
225
150
102
76
260
160
110
80
60

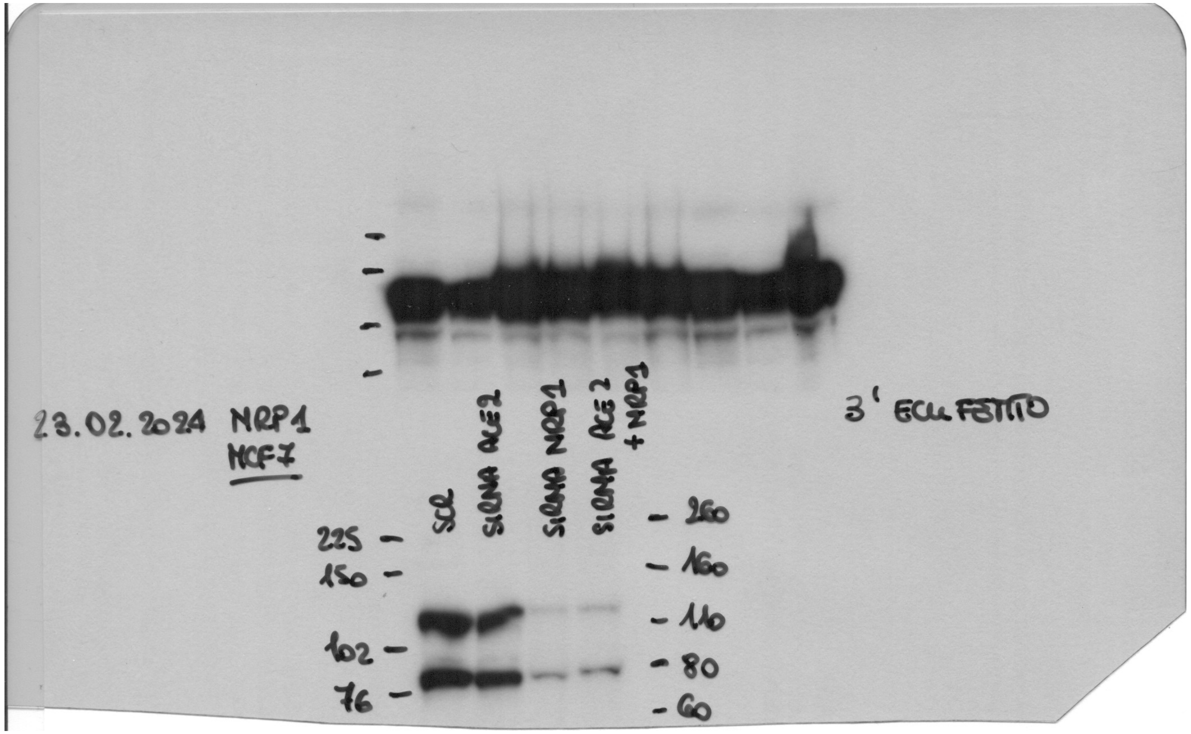
23.02.2024 NRP1
MCF7
3' ECL FATTO
SCR
SIRNA ACE2
SIRNA NRP1
SIRNA ACE2 + NRP1
225
150
102
76
260
160
110
80
60

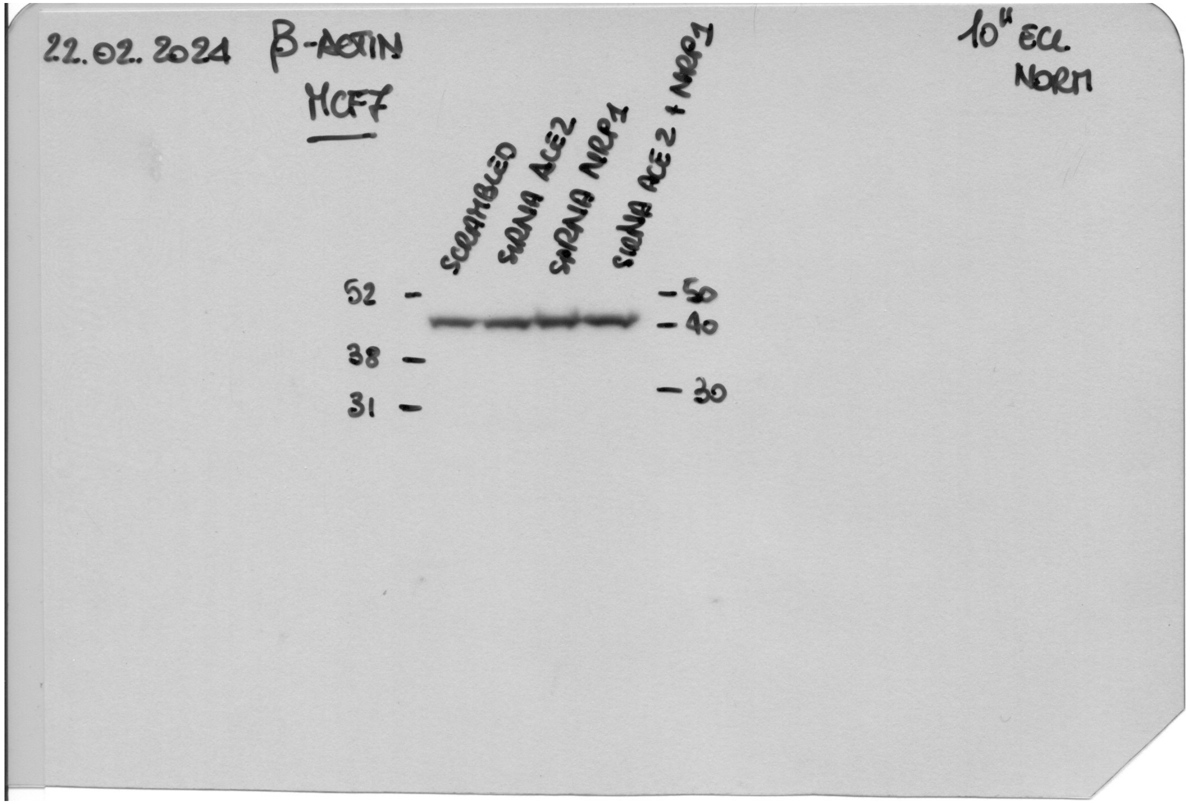
22.02.2024
β-ACTIN
MCF7
10'' ECL
NORM
SCRAMBLED
siRNA ACE2
siRNA NRP1
siRNA ACE2 + NRP1
52
38
31
50
40
30

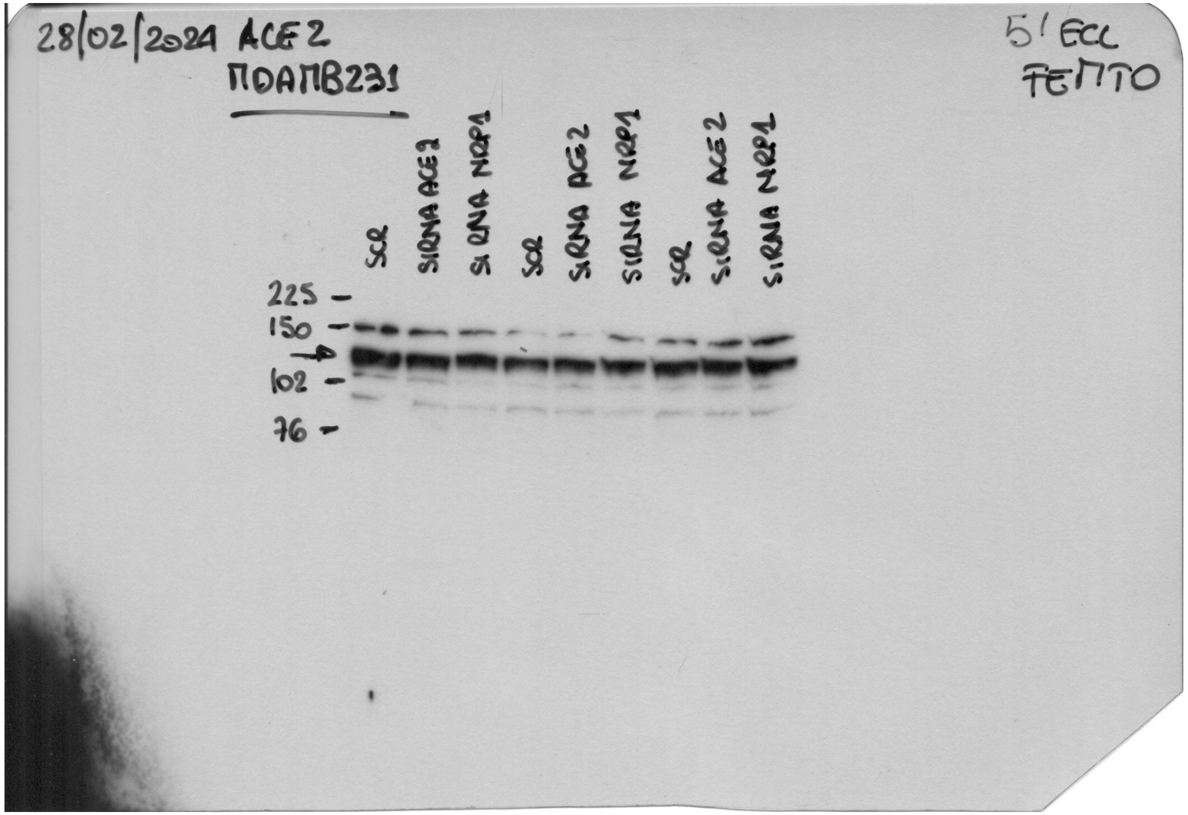
28/02/2024 ACE2
MDAMB231
5' ECL
FEMTO
SCR
SIRNA ACE2
SIRNA NRP1
SCR
SIRNA ACE2
SIRNA NRP1
SCR
SIRNA ACE2
SIRNA NRP1
225
150
102
76

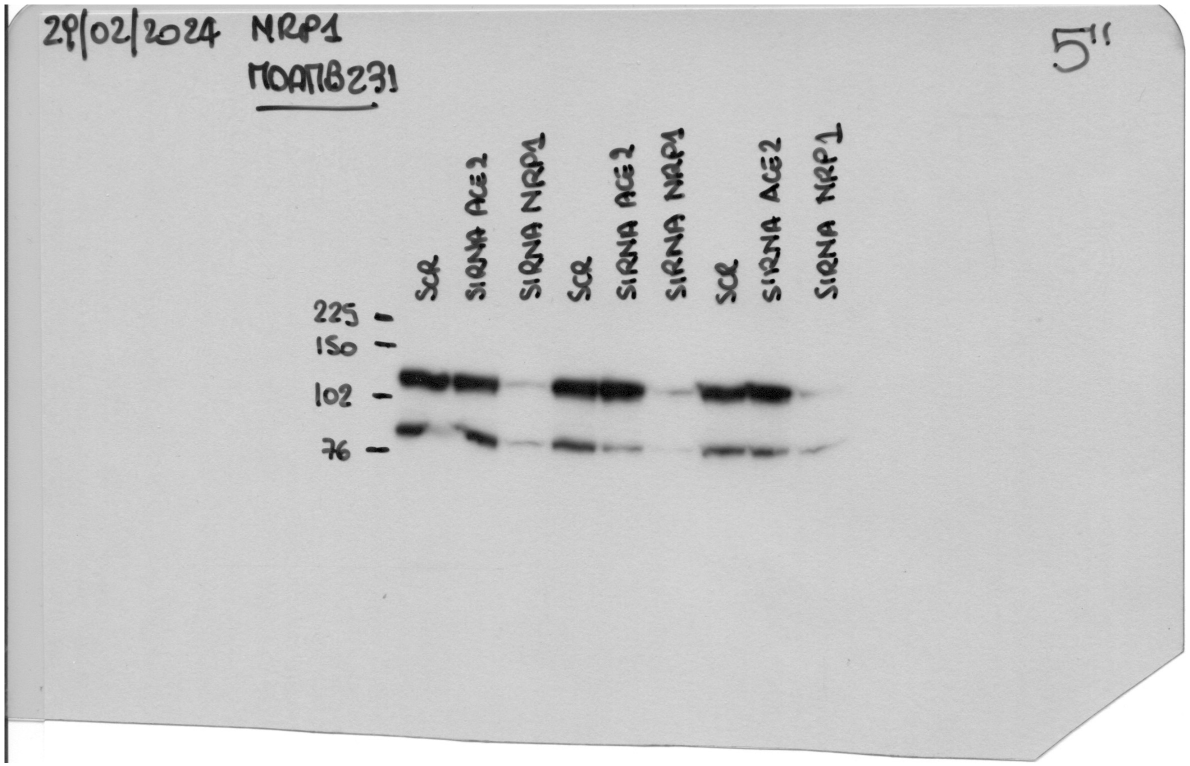
29/02/2024
NRP1
MDAMB231
5''
SCR
SIRNA ACE2
SIRNA NRP1
SCR
SIRNA ACE2
SIRNA NRP1
SCR
SIRNA ACE2
SIRNA NRP1
225
150
102
76

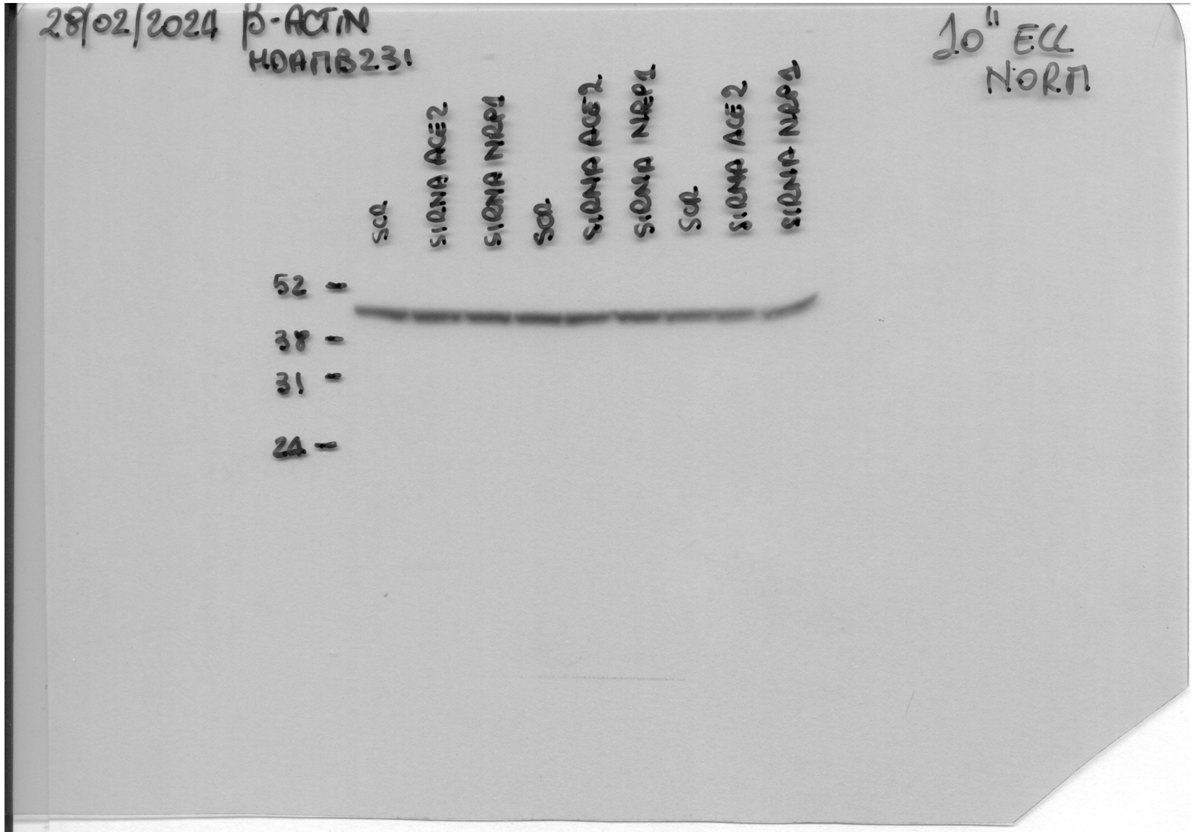

28/02/2024 β-ACTIN
MDAMB231
10'' ECL
NORM
SCR
siRNA ACE2
siRNA NRP1
SCR
siRNA ACE2
siRNA NRP1
SCR
siRNA ACE2
siRNA NRP1
52
38
31
24

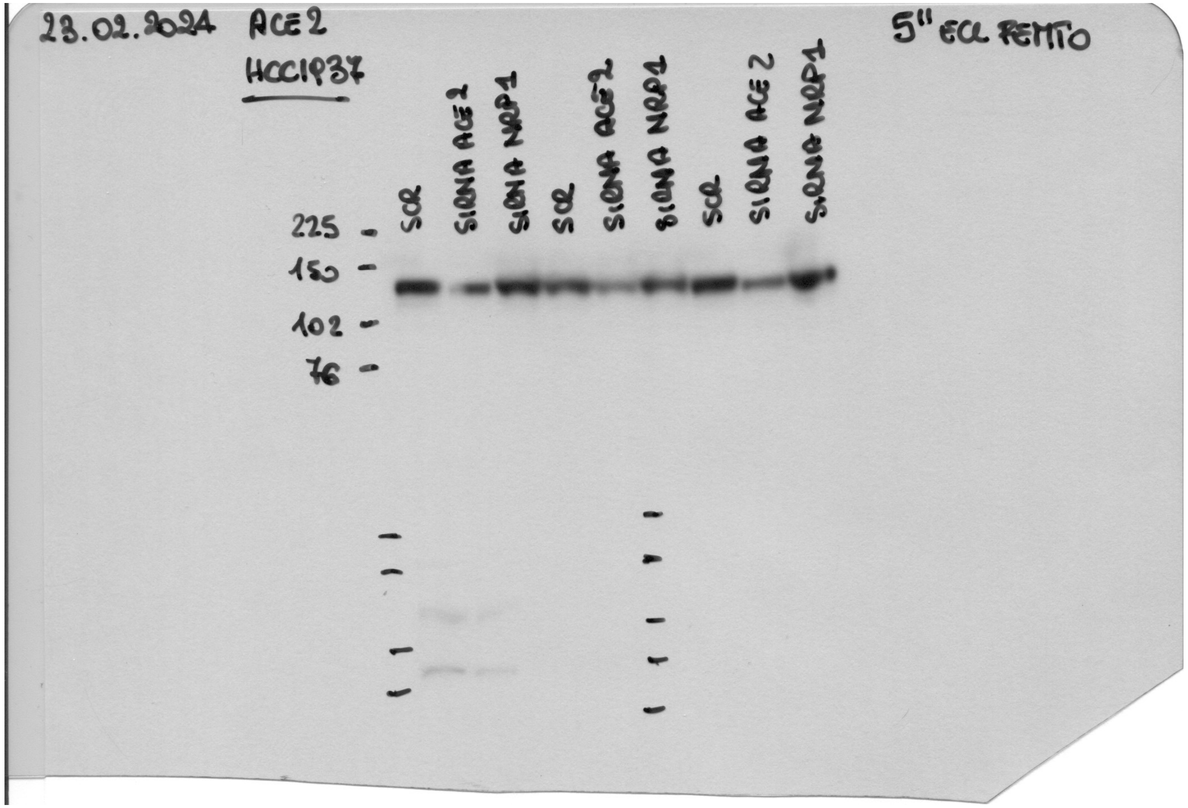
23.02.2024
ACE2
HCC1937
5'' ECL FEMTO
SCR
SIRNA ACE2
SIRNA NRP1
SCR
SIRNA ACE2
SIRNA NRP1
SCR
SIRNA ACE2
SIRNA NRP1
225
150
102
76

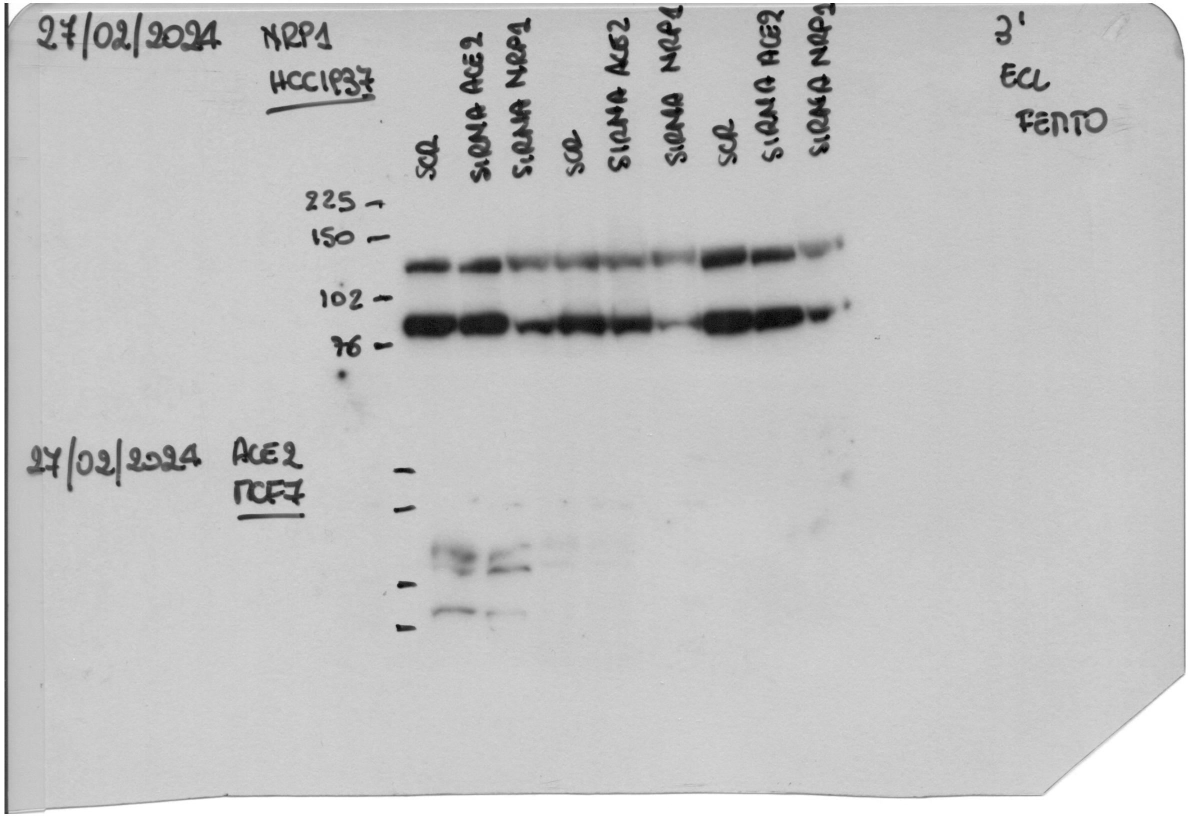
27/02/2024
NRP1
HCC1937
SCR
SIRNA ACE2
SIRNA NRP1
SCR
SIRNA ACE2
SIRNA NRP1
SCR
SIRNA ACE2
SIRNA NRP1
3'
ECL
FEMTO
225
150
102
76
27/02/2024
ACE2
MCF7

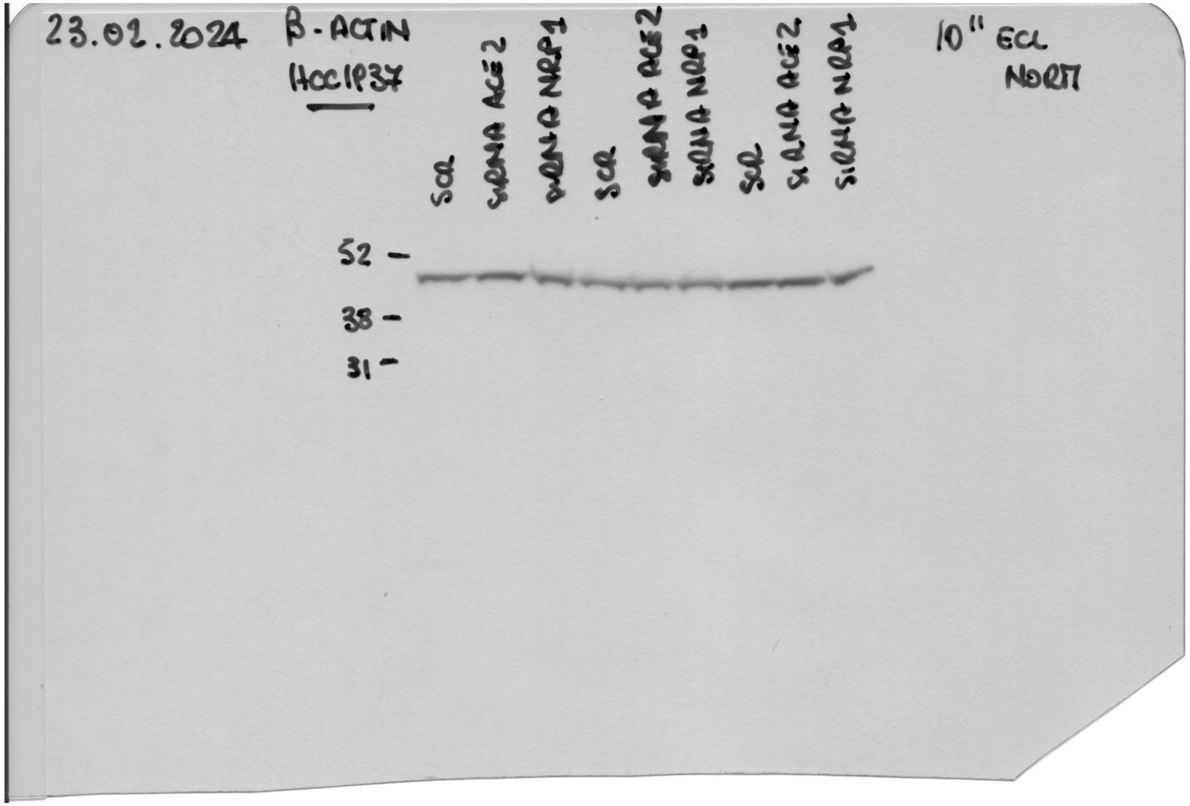
23.02.2024
β-ACTIN
HCC1937
SCR
siRNA ACE2
siRNA NRP1
SCR
siRNA ACE2
siRNA NRP1
SCR
siRNA ACE2
siRNA NRP1
10'' ECL
NORM
52
38
31
